# Publisher Correction: Endophenotype effect sizes support variant pathogenicity in monogenic disease susceptibility genes

**DOI:** 10.1038/s41467-022-33534-z

**Published:** 2022-09-30

**Authors:** Jennifer L. Halford, Valerie N. Morrill, Seung Hoan Choi, Sean J. Jurgens, Giorgio Melloni, Nicholas A. Marston, Lu-Chen Weng, Victor Nauffal, Amelia W. Hall, Sophia Gunn, Christina A. Austin-Tse, James P. Pirruccello, Shaan Khurshid, Heidi L. Rehm, Emelia J. Benjamin, Eric Boerwinkle, Jennifer A. Brody, Adolfo Correa, Brandon K. Fornwalt, Namrata Gupta, Christopher M. Haggerty, Stephanie Harris, Susan R. Heckbert, Charles C. Hong, Charles Kooperberg, Henry J. Lin, Ruth J. F. Loos, Braxton D. Mitchell, Alanna C. Morrison, Wendy Post, Bruce M. Psaty, Susan Redline, Kenneth M. Rice, Stephen S. Rich, Jerome I. Rotter, Peter F. Schnatz, Elsayed Z. Soliman, Nona Sotoodehnia, Eugene K. Wong, NHLBI Trans-Omics for Precision Medicine (TOPMed) Consortium, Marc S. Sabatine, Christian T. Ruff, Kathryn L. Lunetta, Patrick T. Ellinor, Steven A. Lubitz

**Affiliations:** 1 Cardiovascular Disease Initiative, Broad Institute of MIT and Harvard, Cambridge, MA USA; 2 Department of Medicine, Massachusetts General Hospital, Boston, MA USA; 3 Cardiovascular Research Center, Massachusetts General Hospital, Boston, MA USA; 4 Department of Experimental Cardiology, Amsterdam UMC, Amsterdam, The Netherlands; 5 TIMI Study Group, Division of Cardiovascular Medicine, Brigham and Women’s Hospital, Boston, MA USA; 6 Gene Regulation Observatory and Epigenomics Platform, Broad Institute of MIT and Harvard, Cambridge, MA USA; 7 Department of Biostatistics, Boston University School of Public Health, Boston, MA USA; 8 Laboratory for Molecular Medicine, Mass General Brigham Personalized Medicine, Cambridge, MA USA; 9 Harvard Medical School, Boston, MA USA; 10 Center for Genomic Medicine, Massachusetts General Hospital, Boston, MA USA; 11 Demoulas Center for Cardiac Arrhythmias, Massachusetts General Hospital, Boston, MA USA; 12 NHLBI and Boston University’s Framingham Heart Study, Framingham, MA USA; 13 Department of Medicine, Boston Medical Center, Boston University School of Medicine, Boston, MA USA; 14 Department of Epidemiology, Boston University School of Public Health, Boston, MA USA; 15 Human Genetics Center, Department of Epidemiology, Human Genetics, and Environmental Sciences, School of Public Health, The University of Texas Health Science Center at Houston, Houston, TX USA; 16 Cardiovascular Health Research Unit, Department of Medicine, University of Washington, Seattle, WA USA; 17 Departments of Medicine, Pediatrics and Population Health Science, University of Mississippi Medical Center, Jackson, MS USA; 18 Department of Translational Data Science and Informatics, Geisinger, Danville, PA USA; 19 Heart Institute, Geisinger, Danville, PA USA; 20 Department of Radiology, Geisinger, Danville, PA USA; 21 Department of Epidemiology, University of Washington, Seattle, WA USA; 22 University of Maryland School of Medicine, Baltimore, MD USA; 23 Division of Public Health Sciences, Fred Hutchinson Cancer Center, Seattle, WA USA; 24 The Institute for Translational Genomics and Population Sciences, Department of Pediatrics, The Lundquist Institute for Biomedical Innovation at Harbor-UCLA Medical Center, Torrance, CA USA; 25 The Charles Bronfman Institute for Personalized Medicine, Icahn School of Medicine at Mount Sinai, 10029 New York, NY USA; 26 The Mindich Child Health and Development Institute, Icahn School of Medicine at Mount Sinai, 10029 New York, NY USA; 27 Geriatrics Research and Education Clinical Center, Baltimore Veterans Administration Medical Center, Baltimore, MD USA; 28 Division of Cardiology, Johns Hopkins Medicine, Baltimore, MD USA; 29 Department of Health Systems and Population Health, University of Washington, Seattle, WA USA; 30 Department of Medicine, Brigham and Women’s Hospital, Boston, MA USA; 31 Department of Biostatistics, University of Washington, Seattle, WA USA; 32 Center for Public Health Genomics, Department of Public Health Sciences, University of Virginia, Charlottesville, VA USA; 33 Department of ObGyn, The Reading Hospital of Tower Health, Reading, PA USA; 34 Epidemiological Cardiology Research Center, Wake Forest School of Medicine, Winston Salem, NC USA; 35 Division of Cardiology, Department of Medicine, University of Washington, Seattle, WA USA

**Keywords:** Cardiology, Genetic testing, Cardiovascular diseases, Molecular medicine

Correction to: *Nature Communications* 10.1038/s41467-022-32009-5, published online 30 August 2022

The original PDF version of this article contained an error in Figure 2, in which the left-hand edge of the figure was inadvertently cropped, removing the panel labels and portions of the *y*-axis labels. This has been corrected in the PDF version of the article. The HTML version was correct from the time of publication.

