# Supplementary material for: Endophenotype effect sizes support variant pathogenicity in monogenic disease susceptibility genes
Source: Nat Commun. 2022 Aug 30;13:5106. doi: 10.1038/s41467-022-32009-5 (PMC9427940; doi:10.1038/s41467-022-32009-5)
Supplement: Supplementary file 1 — Supplementary Information [file 41467_2022_32009_MOESM1_ESM.pdf]

SUPPLEMENTARY INFORMATION

Items	Page
Supplementary Methods	3
Supplementary Table 1. Baseline replication cohort characteristics by endophenotype	4
Supplementary Table 2. Baseline cohort characteristics by endophenotype in European-ancestry participants of the UK Biobank	5
Supplementary Table 3. Distribution and characteristics of variants in LDL-C associated genetic testing panels of interest	6
Supplementary Table 4. Distribution and characteristics of variants in QTc-associated genetic testing panels of interest	7
Supplementary Table 5. Distribution and characteristics of variants in HbA1c-associated genetic testing panels of interest	8
Supplementary Table 6. Distribution and characteristics of variants in LDL-C associated genetic testing panels of interest in FOURIER	9
Supplementary Table 7. Distribution and characteristics of variants in QTc-associated genetic testing panels of interest in TOPMed	10
Supplementary Table 8. Distribution and characteristics of variants in HbA1c-associated genetic testing panels of interest in FOURIER	11
Supplementary Table 9. Discriminative ability of various effect size thresholds	12
Supplementary Table 10. Variants with large effect sizes observed in ClinVar with uncertain significance or conflicting assertions	13
Supplementary Table 11. Variants with large effect size stratified by variant consequence	14
Supplementary Table 12. Splice-altering predictions of synonymous variants	15
Supplementary Table 13. Genetic testing panels of interest	16
Supplementary Figure 1. CONSORT diagram of LDL-C endophenotype analysis	17
Supplementary Figure 2. CONSORT diagram of QTc endophenotype analysis	18
Supplementary Figure 3. CONSORT diagram of HbA1c endophenotype analysis	19
Supplementary Figure 4. Association between variant effect size and pathogenicity for three endophenotypes and a control panel	20
Supplementary Figure 5. Association between variant effect size and pathogenicity for three endophenotypes in a European-ancestry cohort	21

Supplementary Figure 6. Association between variant effect size and pathogenicity for three endophenotypes in replication cohorts	22
Supplementary Figure 7. Heatmap of bioinformatic tool functional predictions for variants of uncertain significance with large effect sizes	23
Supplementary Figure 8. Heatmap of bioinformatic tool functional predictions for variants with conflicting assertions with large effect sizes	24
Supplementary Figure 9. Proportion of large effect size variants by variant consequence	25
Supplementary Note 1. Funding information	26-29
Supplementary Note 2. TOPMed consortium author list	30-38
Supplementary References	38

Please see separate supplementary Excel file for the following data:
Supplementary Data 1. Characteristics of variants of uncertain significance and conflicting assertions with large effect sizes
Supplementary Data 2. Characteristics of variants not reported in ClinVar with large effect sizes

Supplementary Methods

Estimation of genetic ancestry for TOPMed

To estimate genetic ancestry within the TOPMed cohort, we selected common (MAF ≥ 5%), high confidence (missingness rate <1%) variants in the 1000G dataset that were also present in TOPMed. We pruned these variants within five ethnic groups in the 1000G dataset (European, East Asian, South Asian, Admixed American, and African American) using PLINK ("--indep-pairwise 50 5 0.2).[1] We used ADMIXTURE to learn the genetic structure of the five reference ethnic groups, and then projected the TOPMed participants on the reference groups.[2] We classified TOPMed individuals into one of the five ethnic groups when the probability of being a member of that group was > 80%. Individuals with <80% probability of being a member of all five ethnic groups were classified as "Undetermined".

Clinical covariates and exclusion criteria in the UKBB

Baseline history of myocardial infarction was defined centrally using an algorithmically defined outcome, which takes self-reports, hospital admissions and death records into account.[3] Baseline history of heart failure was defined using: self-reported non-cancer illness codes 1076, 1079 and 1588; ICD10 codes I11.0, I13.0, I.13.2, I25.5, I42.0, I42.1, I42.2, I42.5, I42.8, I42.9, I50, I50.0, I50.1 and I50.9 among main diagnoses, secondary diagnoses, primary causes of death and secondary causes of death; and ICD9 codes 4254, 4280, 4281 and 4289 among main and secondary diagnoses. Presence of cardiac pacemaker was defined using: self-reported presence of pacemaker during interview; self-reported operation codes 1096, 1548 and 1549; ICD10 code Z45.0 among main and secondary diagnoses; operative procedure codes K60, K60.1, K60.2, K60,3, K60.4, K60.5, K60.6, K60.7, K60.8, K60.9, K61, K61.1, K61.2, K61.3, K61.4, K61.5, K61.6, K61.7, K61.8 and K61.9 among main and secondary OPCS. Wolff-Parkinson-White syndrome was defined using: self-reported non-cancer illness code 1484; ICD10 code I45.6 among main diagnoses, secondary diagnoses, primary causes of death and secondary causes of death; ICD9 code 4267 among primary and secondary diagnoses; operative procedure codes K52.4 and K57.4 among main and secondary OPCS. Additionally, for the LDL analysis, HDL was measured by the same Beckman Coulter AU5800 device at the time of LDL measurement. Statin usage was ascertained via self-reported data at initial assessment.

Supplementary Table 1. Baseline replication cohort characteristics by endophenotype

**Characteristic**	**LDL-C (mg/dL)**	**QTc (ms)**	**HbA1c (%)**
Cohort name	FOURIER	TOPMed	FOURIER
Participants with a measurable endophenotype, n	14,038	26,976	12,798
Median endophenotype value (Q1-Q3)	92.0 (80.0-109.0)	421.0 (409.0-437.0)	5.8 (5.5-6.3)
Male, n (%)	10,720 (76.4)	9,332 (34.6)	9,731 (76.0)
European ancestry, n (%)	14,023 (100.0%)	16,074 (59.5)	12,783 (99.8)
Mean age, years (SD)	62.8 (8.8)	59.8 (12.5)	62.9 (8.8)
Myocardial infarction, n (%)	11,470 (81.7)	2,361 (8.8)	-
Statin usage, n (%)	14,039 (100.0)	-	-
Median high-density lipoprotein, mg/dL (Q1-Q3)	45.0 (38.0-53.5)	-	-
Heart failure, n (%)	-	1,788 (6.6)	-
Beta blocker usage, n (%)	-	3,415 (12.6)	-
Calcium channel blocker usage, n (%)	-	3,043 (11.3)	-
Type 2 diabetes medication usage, n (%)	-	-	3,265 (25.6)
Mean corpuscular volume, femtoliters (SD)	-	-	92.8 (4.8)
NB: only select relevant characteristics for the given monogenic disease of interest are displayed<br>This table includes median imputed values for select clinical covariates			

Supplementary Table 2. Baseline cohort characteristics by endophenotype in European-ancestry participants of the UK Biobank

Characteristic	LDL-C (mg/dL)	QTc (ms)	HbA1c (%)
Participants with a measurable endophenotype, n	165,783	28,249	166,335
Median endophenotype value (Q1-Q3)	136.6 (114.5-159.6)	411.3 (396.4-426.4)	5.4 (5.1-5.6)
Male, n (%)	74,747 (45.1)	14,138 (50.0)	74,983 (45.0)
Mean age, years (SD)	57.3 (8.0)	53.0 (5.6)	57.3 (8.0)
Myocardial infarction, n (%)	2,668 (1.6)	422 (1.5)	-
Statin usage, n (%)	27,796 (16.8)	-	-
Median high-density lipoprotein, mg/dL (Q1-Q3)	54.6 (46.5-64.1)	-	-
Heart failure, n (%)	-	66 (0.2)	-
Beta blocker usage, n (%)	-	1,490 (5.3)	-
Calcium channel blocker usage, n (%)	-	1,968 (7.0)	-
Type 2 diabetes medication usage, n (%)	-	-	5,700 (3.4)
Mean corpuscular volume, femtoliters (SD)	-	-	91.4 (4.2)
NB: only select relevant characteristics for the given monogenic disease of interest are displayed<br>This table includes median imputed values for select clinical covariates			

Supplementary Table 3. Distribution and characteristics of variants in LDL-C associated genetic testing panels of interest

	**Definitive FH genes**	**Commercially available hereditary cancer panel**
Total variants in SVA	3,544	25,323
Variants of MAF < 0.1%	3,495	24,984
Variants of MAF < 0.1% not in ClinVar	2,052	7,719
Variants of MAF < 0.1% in ClinVar	1,443	17,265
Pathogenic (P) variants	45	757
Mean effect size, mg/dL(SD)	46.57 (52.58)	-0.52 (24.60)
Median MAC (Q1-Q3)	2 (1-5)	2 (1-4)
Likely pathogenic (LP) variants	18	164
Mean effect size, mg/dL(SD)	44.50 (58.43)	-2.51 (24.06)
Median MAC (Q1-Q3)	2 (1-2)	2 (1-4)
Likely benign (LB) variants	417	4,378
Mean effect size, mg/dL(SD)	-2.13 (21.36)	0.18 (21.91)
Median MAC (Q1-Q3)	4 (2-11)	2 (1-6)
Benign (B) variants	44	921
Mean effect size, mg/dL(SD)	-0.77 (12.94)	0.03 (12.17)
Median MAC (Q1-Q3)	19 (6.75-51.75)	23 (6-67)
Variants of uncertain significance (VUS)	624	8,789
Mean effect size, mg/dL(SD)	-2.06 (22.01)	0.58 (23.17)
Median MAC (Q1-Q3)	4 (2-10)	2 (1-4)
Variants of conflicting interpretations (C)	295	2,256
Mean effect size, mg/dL(SD)	4.83 (20.53)	-0.08 (16.55)
Median MAC (Q1-Q3)	12 (4-38)	7 (2-19)

FH: Familial Hypercholesterolemia, SVA: single variant association, MAF: minor allele frequency, B: benign, LB: likely benign, LP: likely pathogenic, P: pathogenic, VUS: variant of uncertain significance, C: conflicting

Supplementary Table 4. Distribution and characteristics of variants in QTc-associated genetic testing panels of interest

	**Definitive LQTS genes**	**Commercially available hereditary cancer panel**
Total variants in SVA	1,104	11,144
Variants of MAF < 0.1%	1,078	10,769
Variants of MAF < 0.1% not in ClinVar	326	2,395
Variants of MAF < 0.1% in ClinVar	752	8,374
Pathogenic (P) variants	20	286
Mean effect size, ms (SD)	29.58 (29.19)	1.03 (19.74)
Median MAC (Q1-Q3)	1 (1-2)	1 (1-2)
Likely pathogenic (LP) variants	13	64
Mean effect size, ms (SD)	11.54 (36.98)	-1.64 (26.68)
Median MAC (Q1-Q3)	2 (1-2)	1 (1-2)
Likely benign (LB) variants	230	2,125
Mean effect size, ms (SD)	1.76 (16.99)	0.56 (19.34)
Median MAC (Q1-Q3)	2 (1-4)	1 (1-3)
Benign (B) variants	41	740
Mean effect size, ms (SD)	-0.74 (11.84)	-0.04 (13.03)
Median MAC (Q1-Q3)	7 (3-13)	6 (2-17)
Variants of uncertain significance (VUS)	279	3,681
Mean effect size, ms (SD)	0.00 (18.38)	-0.49 (19.28)
Median MAC (Q1-Q3)	1 (1-2.5)	1 (1-2)
Variants of conflicting interpretations (C)	169	1,478
Mean effect size, ms (SD)	0.95 (16.12)	-0.05 (16.10)
Median MAC (Q1-Q3)	3 (1-8)	3 (1-7)

LQTS: Long-QT syndrome, SVA: single variant association, MAF: minor allele frequency, B: benign, LB: likely benign, LP: likely pathogenic, P: pathogenic, VUS: variant of uncertain significance, C: conflicting

Supplementary Table 5. Distribution and characteristics of variants in HbA1c-associated genetic testing panels of interest

	**Common MODY genes**	**Commercially available hereditary cancer panel**
Total variants in SVA	1,062	24,748
Variants of MAF < 0.1%	1,052	24,411
Variants of MAF < 0.1% not in ClinVar	868	7,191
Variants of MAF < 0.1% in ClinVar	184	17,220
Pathogenic (P) variants	20	757
Mean effect size, % (SD)	0.70 (0.70)	0.01 (0.38)
Median MAC (Q1-Q3)	1.5 (1-2)	2 (1-4)
Likely pathogenic (LP) variants	6	162
Mean effect size, % (SD)	0.45 (0.37)	-0.03 (0.28)
Median MAC (Q1-Q3)	1 (1-1.75)	2 (1-4)
Likely benign (LB) variants	29	4,338
Mean effect size, % (SD)	-0.05 (0.19)	-0.01 (0.39)
Median MAC (Q1-Q3)	11 (4-33)	2 (1-6)
Benign (B) variants	28	931
Mean effect size, % (SD)	0.05 (0.20)	0.00 (0.21)
Median MAC (Q1-Q3)	30.5 (14.25-70.75)	22 (6-62)
Variants of uncertain significance (VUS)	67	8,775
Mean effect size, % (SD)	0.01 (0.27)	0.00 (0.43)
Median MAC (Q1-Q3)	4 (2-16)	2 (1-4)
Variants of conflicting interpretations (C)	34	2,257
Mean effect size, % (SD)	0.04 (0.23)	0.00 (0.33)
Median MAC (Q1-Q3)	11 (5.25-33.75)	7 (2-18)

MODY: maturity-onset diabetes of the young, SVA: single variant association, MAF: minor allele frequency, B: benign, LB: likely benign, LP: likely pathogenic, P: pathogenic, VUS: variant of uncertain significance, C: conflicting

Supplementary Table 6. Distribution and characteristics of variants in LDL-C associated genetic testing panels of interest in FOURIER

	**Definitive FH genes**	**Commercially available hereditary cancer panel**
Total variants in SVA	898	5,402
Variants of MAF < 0.1%	855	5,105
Variants of MAF < 0.1% not in ClinVar	324	873
Variants of MAF < 0.1% in ClinVar	531	4,232
Pathogenic (P) variants	58	143
Mean effect size, mg/dL(SD)	61.19 (58.15)	4.21 (30.36)
Median MAC (Q1-Q3)	1 (1-2)	1 (1-1.5)
Likely pathogenic (LP) variants	19	33
Mean effect size, mg/dL(SD)	36.71 (57.75)	-0.31 (19.45)
Median MAC (Q1-Q3)	1 (1-1.5)	1 (1-1)
Likely benign (LB) variants	101	970
Mean effect size, mg/dL(SD)	2.70 (21.99)	-0.42 (23.56)
Median MAC (Q1-Q3)	1 (1-2)	1 (1-2)
Benign (B) variants	18	439
Mean effect size, mg/dL(SD)	-5.39 (13.12)	1.33 (20.54)
Median MAC (Q1-Q3)	2 (1-3)	2 (1-5)
Variants of uncertain significance (VUS)	192	1,827
Mean effect size, mg/dL(SD)	-0.25 (22.98)	0.91 (30.70)
Median MAC (Q1-Q3)	1 (1-2)	1 (1-1)
Variants of conflicting interpretations (C)	143	820
Mean effect size, mg/dL(SD)	9.63 (48.65)	-0.13 (19.56)
Median MAC (Q1-Q3)	2 (1-4.5)	2 (1-4)

FH: Familial Hypercholesterolemia, SVA: single variant association, MAF: minor allele frequency, B: benign, LB: likely benign, LP: likely pathogenic, P: pathogenic, VUS: variant of uncertain significance, C: conflicting

Supplementary Table 7. Distribution and characteristics of variants in QTc-associated genetic testing panels of interest in TOPMed

	**Definitive LQTS genes**	**Commercially available hereditary cancer panel**
Total variants in SVA	1,190	11,915
Variants of MAF < 0.1%	1,131	11,247
Variants of MAF < 0.1% not in ClinVar	324	2,117
Variants of MAF < 0.1% in ClinVar	807	9,130
Pathogenic (P) variants	23	280
Mean effect size, ms (SD)	37.20 (35.94)	2.68 (21.01)
Median MAC (Q1-Q3)	1 (1-1.5)	1 (1-2)
Likely pathogenic (LP) variants	12	65
Mean effect size, ms (SD)	11.61 (34.24)	0.75 (20.32)
Median MAC (Q1-Q3)	1 (1-2.25)	1 (1-2)
Likely benign (LB) variants	240	2,380
Mean effect size, ms (SD)	1.51 (25.35)	0.39 (18.59)
Median MAC (Q1-Q3)	2 (1-3)	1 (1-3)
Benign (B) variants	33	617
Mean effect size, ms (SD)	2.68 (14.12)	0.10 (13.09)
Median MAC (Q1-Q3)	5 (1-24)	5 (2-13)
Variants of uncertain significance (VUS)	302	4,143
Mean effect size, ms (SD)	-0.85 (21.32)	-0.33 (19.71)
Median MAC (Q1-Q3)	1 (1-3)	1 (1-2)
Variants of conflicting interpretations (C)	197	1,645
Mean effect size, ms (SD)	0.86 (15.44)	0.54 (15.82)
Median MAC (Q1-Q3)	3 (2-7)	2 (1-6)

LQTS: Long-QT syndrome, SVA: single variant association, MAF: minor allele frequency, B: benign, LB: likely benign, LP: likely pathogenic, P: pathogenic, VUS: variant of uncertain significance, C: conflicting

Supplementary Table 8. Distribution and characteristics of variants in HbA1c-associated genetic testing panels of interest in FOURIER

	**Common MODY genes**	**Commercially available hereditary cancer panel**
Total variants in SVA	195	5,078
Variants of MAF < 0.1%	185	4,775
Variants of MAF < 0.1% not in ClinVar	134	812
Variants of MAF < 0.1% in ClinVar	51	3,963
Pathogenic (P) variants	2	132
Mean effect size, ms (SD)	1.69 (0.55)	0.08 (0.89)
Median MAC (Q1-Q3)	1 (1-1)	1 (1-1)
Likely pathogenic (LP) variants	4	30
Mean effect size, ms (SD)	-0.06 (1.05)	0.18 (0.80)
Median MAC (Q1-Q3)	1 (1-1)	1 (1-1)
Likely benign (LB) variants	10	909
Mean effect size, ms (SD)	0.37 (1.02)	0.02 (0.86)
Median MAC (Q1-Q3)	2 (1-4)	1 (1-2)
Benign (B) variants	6	421
Mean effect size, ms (SD)	-0.17 (0.34)	0.01 (0.69)
Median MAC (Q1-Q3)	2 (1.25-2)	2 (1-4)
Variants of uncertain significance (VUS)	17	1,693
Mean effect size, ms (SD)	0.09 (0.94)	0.02 (0.86)
Median MAC (Q1-Q3)	2 (1-4)	1 (1-1)
Variants of conflicting interpretations (C)	12	778
Mean effect size, ms (SD)	-0.20 (0.76)	0.01 (0.72)
Median MAC (Q1-Q3)	1 (1-3.5)	2 (1-3)

MODY: maturity-onset diabetes of the young, SVA: single variant association, MAF: minor allele frequency, B: benign, LB: likely benign, LP: likely pathogenic, P: pathogenic, VUS: variant of uncertain significance, C: conflicting

Supplementary Table 9. Discriminative ability of various effect size thresholds

	LDL-C					QTc					HbA1c				
SD of trait distribution in the UK Biobank	Effect size (mg/dL)	Sensitivity[#],% (95% CI)	Specificity[#],% (95% CI)	PPV,% (95% CI)	NPV,% (95% CI)	Effect size (ms)	Sensitivity,% (95% CI)	Specificity,% (95% CI)	PPV,% (95% CI)	NPV,% (95% CI)	Effect size (%)	Sensitivity,% (95% CI)	Specificity,% (95% CI)	PPV,% (95% CI)	NPV,% (95% CI)
0.125	4.18	82 (71, 94)	67 (62, 71)	19 (14, 25)	98 (96, 99)	2.97	85 (69, 100)	60 (54,66)	14 (8, 20)	98 (96, 100)	0.08	80 (62, 98)	77 (66, 88)	55 (37, 73)	92 (84, 99)
0.25	8.36	80 (68, 92)	75 (71, 79)	24 (17, 30)	97 (96, 99)	5.93	85 (69, 100)	66 (61, 72)	16 (9, 23)	98 (97, 100)	0.15	80 (62, 98)	88 (79, 96)	70 (51, 88)	93 (86, 100)
0.50*	16.73	76 (63, 88)	88 (85, 91)	37 (27, 47)	97 (96, 99)	11.86	80 (62, 98)	74 (69, 79)	19 (10, 27)	98 (96, 100)	0.31	70 (50, 90)	95 (89, 100)	82 (64, 100)	90 (82, 98)
0.75	25.09	69 (55, 82)	95 (93, 97)	56 (43, 69)	97 (95, 98)	17.80	75 (56, 94)	85 (80, 89)	26 (15, 38)	98 (96, 100)	0.46	65 (44, 86)	98 (95, 100)	93 (79, 100)	89 (81, 97)
1.00	33.45	64 (50, 78)	97 (95, 99)	67 (53, 81)	97 (95, 98)	23.73	55 (33, 77)	92 (88, 95)	32 (17, 48)	96 (94, 99)	0.61	65 (44, 86)	98 (95, 100)	93 (79, 100)	89 (81, 97)

**A priori* determined threshold

[#]Sensitivity and specificity are properties of the effect size threshold's ability to discriminate ClinVar designated pathogenic variants from non-pathogenic variants (likely benign and benign).

SD: standard deviation, PPV: positive predictive value, NPV: negative predictive value

Supplementary Table 10. Variants with large effect sizes observed in ClinVar with uncertain significance or conflicting assertions

Endophenotype	Cohort	Large effect size variants, n (%)	Carriers of large effect size variants, n (%)
LDL	UKBB	155 (16.9)	703 (4.7)
	FOURIER	59 (19.4)	113 (16.0)
QTc	UKBB	96 (21.4)	207 (11.3)
	TOPMed	95 (19.0)	250 (12.1)
HbA1c	UKBB	10 (9.9)	39 (1.5)
	FOURIER	7 (24.1)	11 (11.0)

Large effect size refers to variants with effect size > 0.5 standard deviations of the endophenotype distribution in the UK Biobank. Variants observed in ClinVar with uncertain significance (VUS) or conflicting assertions (C) are included in this table.

Supplementary Table 11. Variants with large effect size stratified by variant consequence

SD threshold	0.5						1.0					
Variant consequence	Loss of function, n		Missense and indel, n		Synonymous, n		Loss of function, n		Missense and indel, n		Synonymous, n	
Effect size	Large	Small	Large	Small	Large	Small	Large	Small	Large	Small	Large	Small
Not in ClinVar	22	91	551	1,961	15	98	15	98	263	2,222	101	985
Overall	79	128	1,054	3,728	60	147	60	147	517	4,248	182	2,105

Loss of function includes frameshift, stop-gained, and splice altering variants. Large effect size refers to variants with effect size > 0.5 standard deviations of the endophenotype distribution in the UK Biobank.

Supplementary Table 12. Splice-altering predictions of synonymous variants

SpliceAI score	Delta score ≥ 0.2, n		Delta score ≥ 0.5, n		Delta score ≥ 0.8, n	
Effect size	Large	Small	Large	Small	Large	Small
Not in ClinVar	5	25	1	6	0	3
Overall	14	54	6	14	1	5

Suggested SpliceAI cutoffs in order of increased probability of splice-altering effect are 0.2 (high recall), 0.5 (recommended), and 0.8 (high precision). Large effect size refers to variants with effect size > 0.5 standard deviations of the endophenotype distribution in the UK Biobank.

Supplementary Table 13. Genetic testing panels of interest

Gene panel name	Number of genes	Genes included
Definitive FH genes	3	*LDLR, APOB, PCSK9*
Definitive LQTS genes	3	*KCNQ1, KCNH2, SCN5A*
Common MODY genes	4	*GCK, HNF1A, HNF1B, HNF4A*
Commercially available hereditary cancer panel (control)	47	*APC, ATM, AXIN2, BARD1, BMPR1A, BRCA1, BRCA2, BRIP1, CDH1, CDK4, CDKN2A, CHEK2, CTNNA1, DICER1, EPCAM, GREM1**[#]*, HOXB13**[#]*, KIT, MEN1, MLH1, MSH2, MSH3, MSH6, MUTYH, NBN, NF1, NTHL1, PALB2, PDGFRA, PMS2, POLD1, POLE, PTEN, RAD50, RAD51C, RAD51D, SDHA, SDHB, SDHC, SDHD, SMAD4, SMARCA4, STK11, TP53, TSC1, TSC2, VHL*
[#]No variants from ClinVar were recovered from this gene in UK Biobank analyses<br>*No variants from ClinVar were recovered from this gene in replication analyses		

FH: Familial hypercholesterolemia
LQTS: Long-QT syndrome
MODY: Maturity-onset diabetes of the young

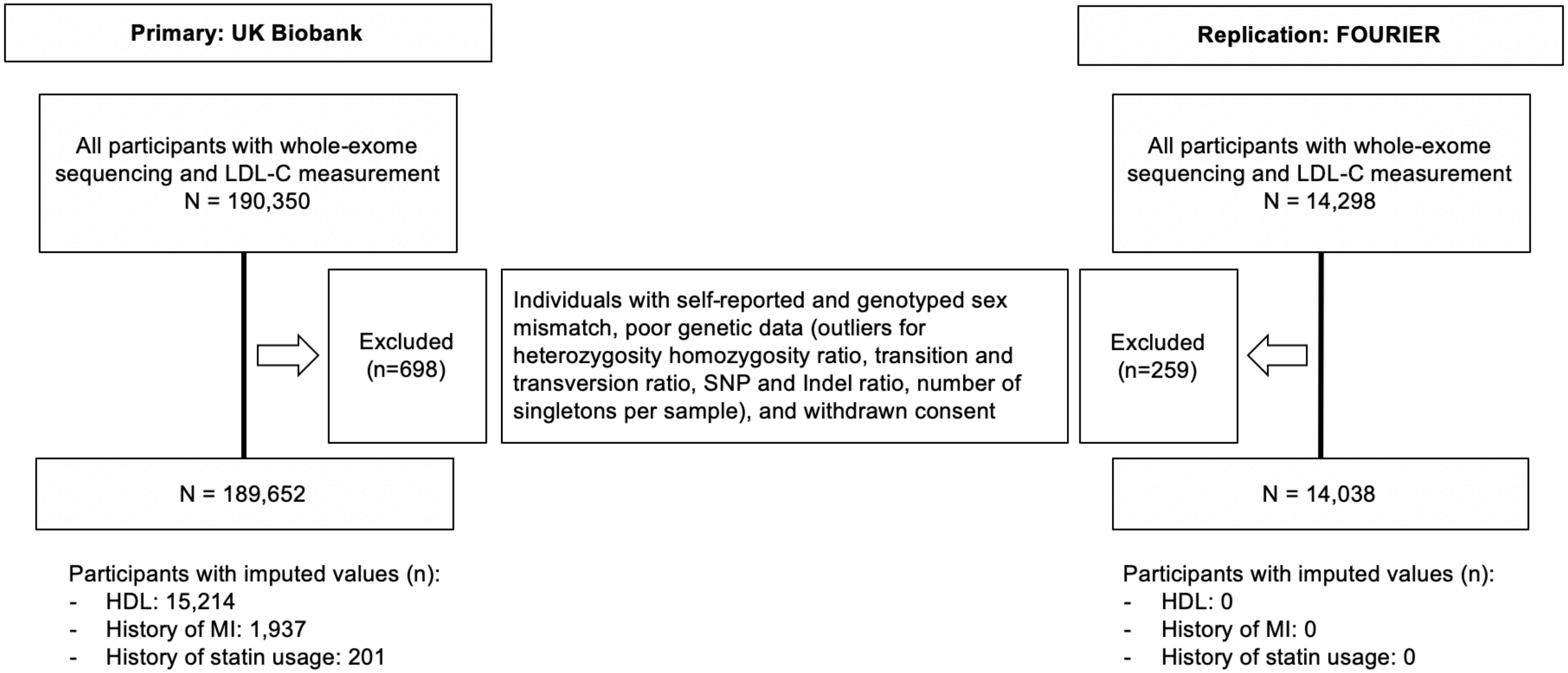

Supplementary Figure 1. CONSORT diagram of LDL-C endophenotype analysis. Each row from top to bottom shows the numbers of individuals excluded at each step. The individuals excluded from the UKBB cohort are on the left, and from the FOURIER cohort are on the right.

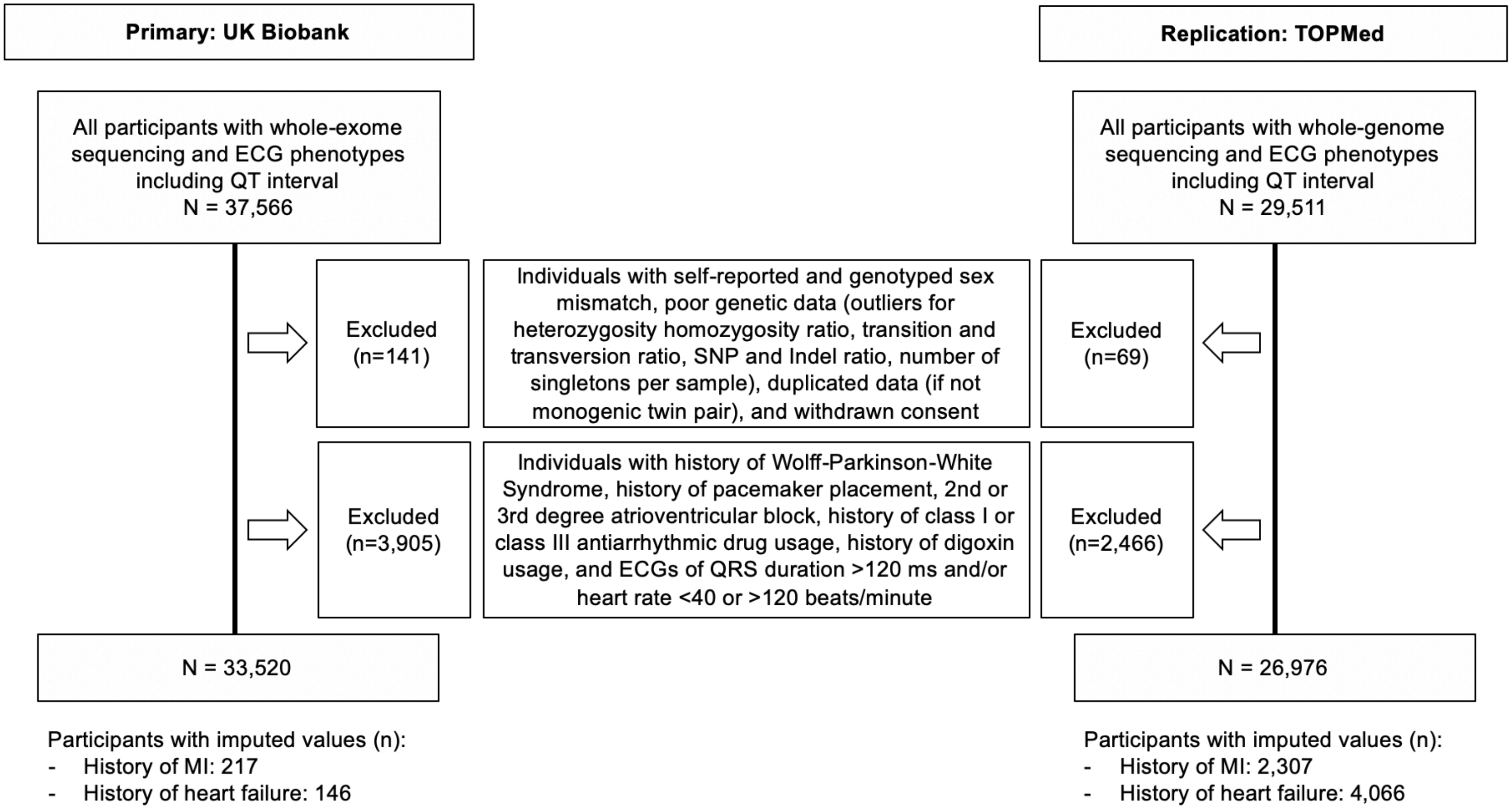

Supplementary Figure 2. **CONSORT diagram of QTc endophenotype analysis.** Each row from top to bottom shows the numbers of individuals excluded at each step. The individuals excluded from the UKBB cohort are on the left, and from the TOPMed cohort are on the right.

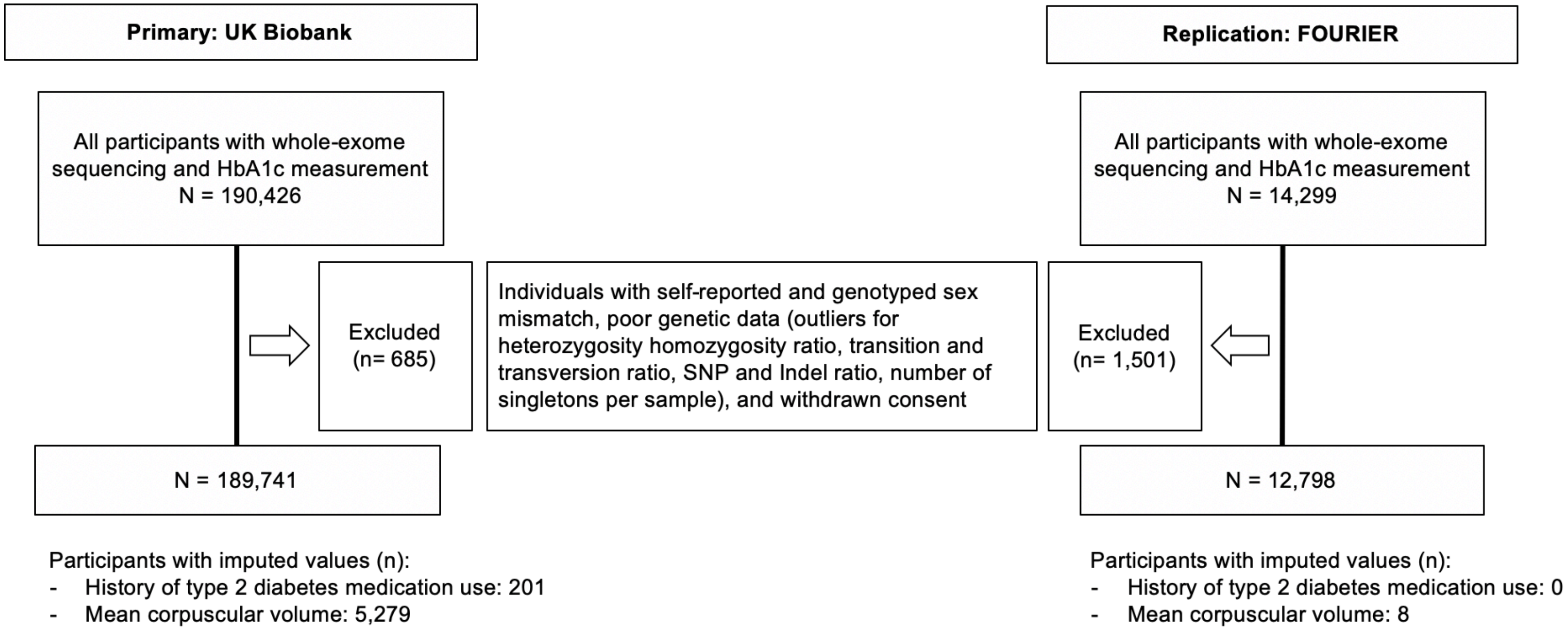

Supplementary Figure 3. CONSORT diagram of HbA1c endophenotype analysis. Each row from top to bottom shows the numbers of individuals excluded at each step. The individuals excluded from the UKBB cohort are on the left, and from the FOURIER cohort are on the right.

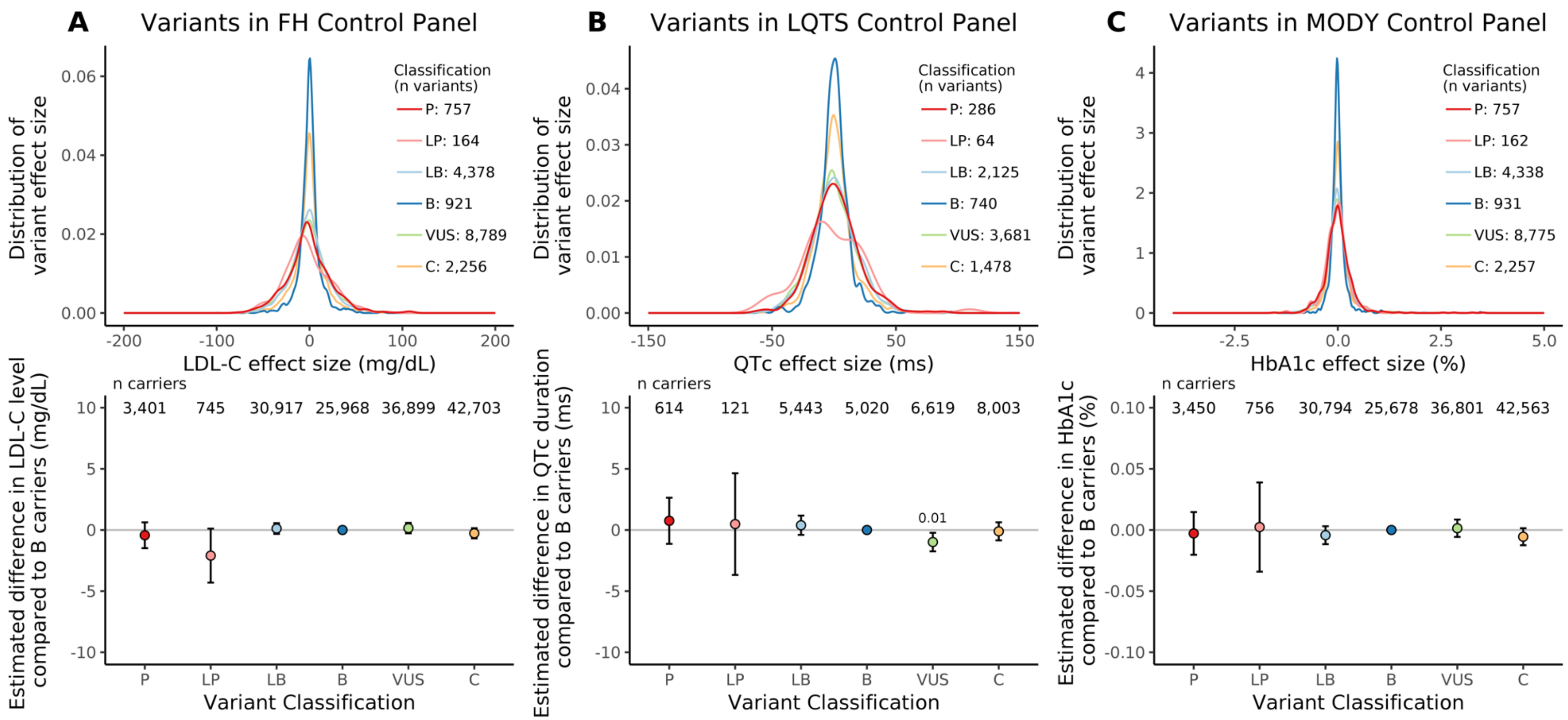

Supplementary Figure 4. Association between variant effect size and pathogenicity for three endophenotypes and a control panel. Association between effect size and variant pathogenicity is shown for three monogenic disease endophenotypes in the control panel of hereditary cancer genes. Panels A, B, and C display data for the LDL-C, QTc, and HbA1c endophenotypes, respectively, for rare variants found in the UK Biobank. Row 1 in each panel displays the variant effect size distribution by ClinVar pathogenicity category (colored). Row 2 in each panel displays the estimated difference in endophenotype value comparing carriers of a variant in each ClinVar pathogenicity category to carriers of benign variants (circles) and 95% confidence intervals. Two-sided P values are derived from multiple linear regression model t-statistics and are annotated for variant categories with $P < 0.05$. Variant classification includes B: benign, LB: likely benign, LP: likely pathogenic, P: pathogenic, VUS: variant of uncertain significance, C: conflicting.

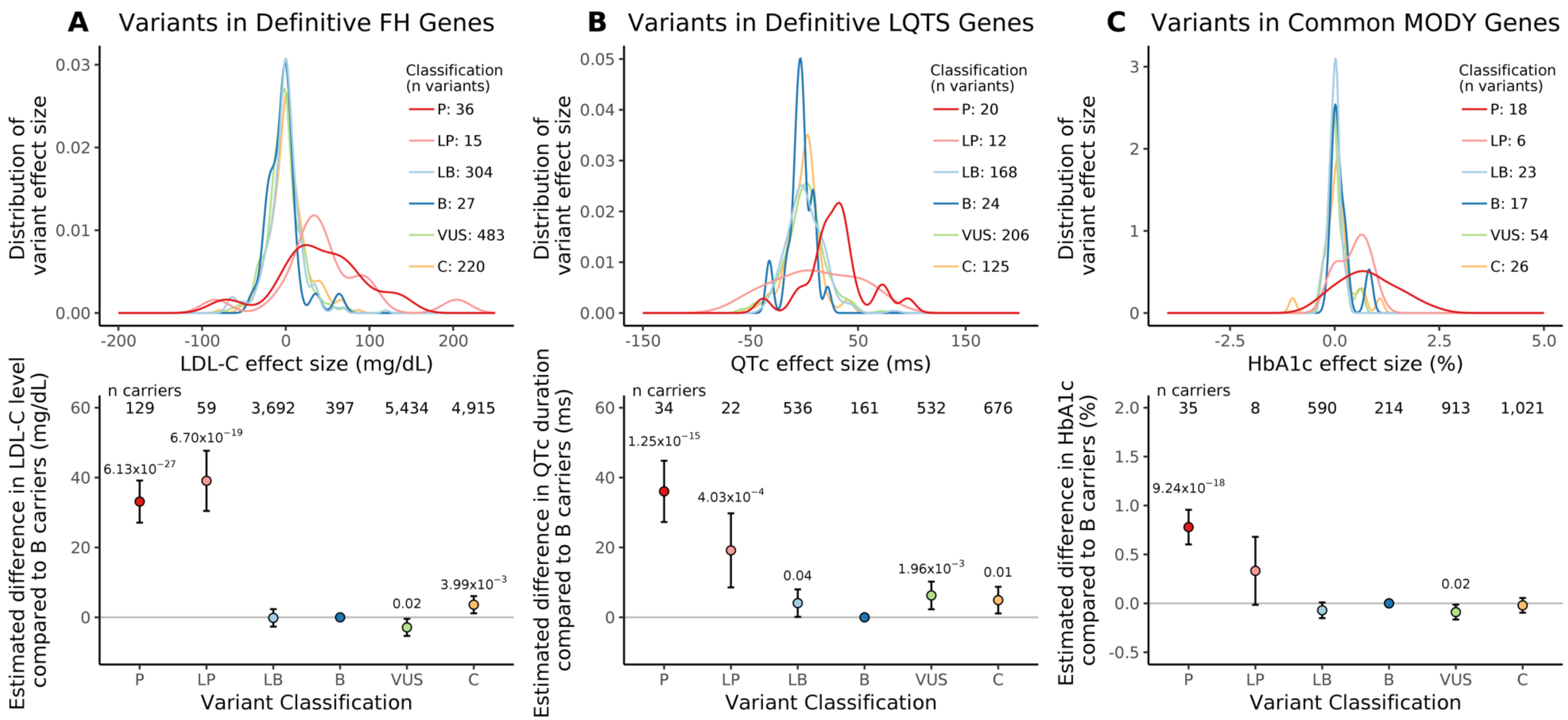

Supplementary Figure 5. Association between variant effect size and pathogenicity for three endophenotypes in a European-ancestry cohort. Association between effect size and variant pathogenicity is shown for three monogenic disease endophenotypes for rare variants found in participants of white European ancestry in the UK Biobank. Panels A, B, and C display data for the LDL-C, QTc, and HbA1c endophenotypes, respectively, for rare variants found in the UK Biobank. Definitive familial hypercholesterolemia (FH) genes include *LDLR, APOB, PCSK9*; definitive long-QT syndrome (LQTS) genes include *KCNQ1, KCNH2, SCN5A*; common maturity-onset diabetes of the young (MODY) genes include *HNF1A, HNF1B, HNF4A, GCK*. Row 1 in each panel displays the variant effect size distribution by ClinVar pathogenicity category (colored). Row 2 in each panel displays the estimated difference in endophenotype value comparing carriers of a variant in each ClinVar pathogenicity category to carriers of benign variants (circles) and 95% confidence intervals. Two-sided P values are derived from multiple linear regression model t-statistics and are annotated for variant categories with $P < 0.05$. Variant classification includes B: benign, LB: likely benign, LP: likely pathogenic, P: pathogenic, VUS: variant of uncertain significance, C: conflicting

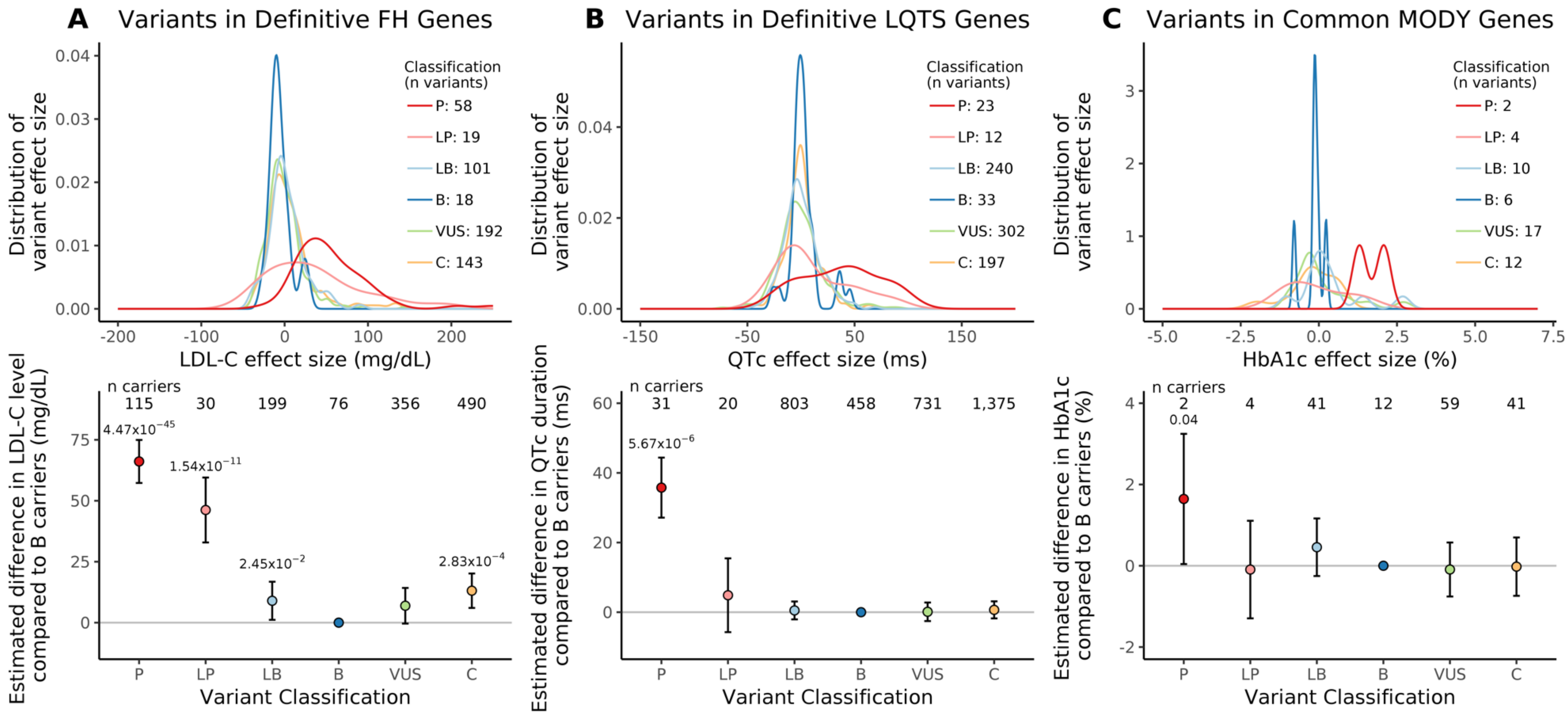

Supplementary Figure 6. **Association between variant effect size and pathogenicity for three endophenotypes in replication cohorts.** Panels A, B, and C display data for the LDL-C, QTc, and HbA1c endophenotypes, respectively, for rare variants found in the FOURIER, TOPMed, and FOURIER, respectively. Definitive familial hypercholesterolemia (FH) genes include *LDLR, APOB, PCSK9*; definitive long-QT syndrome (LQTS) genes include *KCNQ1, KCNH2, SCN5A*; common maturity-onset diabetes of the young (MODY) genes include *HNF1A, HNF1B, HNF4A, GCK*. Row 1 in each panel displays the variant effect size distribution by ClinVar pathogenicity category (colored). Row 2 in each panel displays the estimated difference in endophenotype value comparing carriers of a variant in each ClinVar pathogenicity category to carriers of benign variants (circles) and 95% confidence intervals. Two-sided P values are derived from multiple linear regression model t-statistics and are annotated for variant categories with $P < 0.05$. Variant classification includes B: benign, LB: likely benign, LP: likely pathogenic, P: pathogenic, VUS: variant of uncertain significance, C: conflicting.

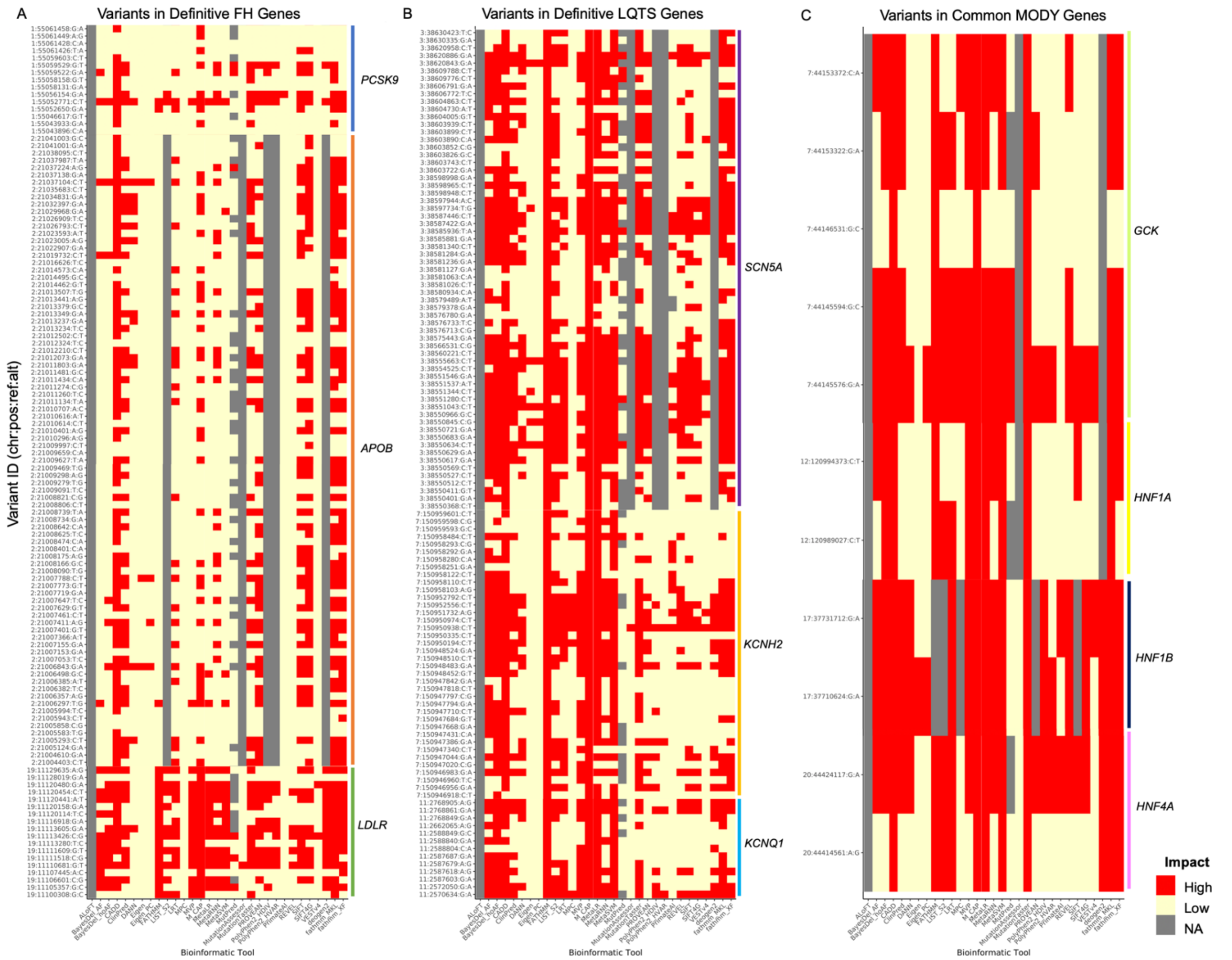

Supplementary Figure 7. Heatmap of bioinformatic tool functional predictions for variants of uncertain significance with large effect sizes. Red boxes represent high predicted functional impact, yellow boxes represent low predicted functional impact, and grey boxes represent no prediction available. Panels A, B, and C display data for rare variants associated with LDL-C, QTc, and HbA1c endophenotypes, respectively. Definitive familial hypercholesterolemia (FH) genes include *LDLR, APOB, PCSK9*. Definitive long-QT syndrome (LQTS) genes include *KCNQ1, KCNH2, SCN5A*. Common maturity-onset diabetes of the young (MODY) genes include *HNF1A, HNF1B, HNF4A, GCK*; the color bar indicates the corresponding gene for the variants shown.

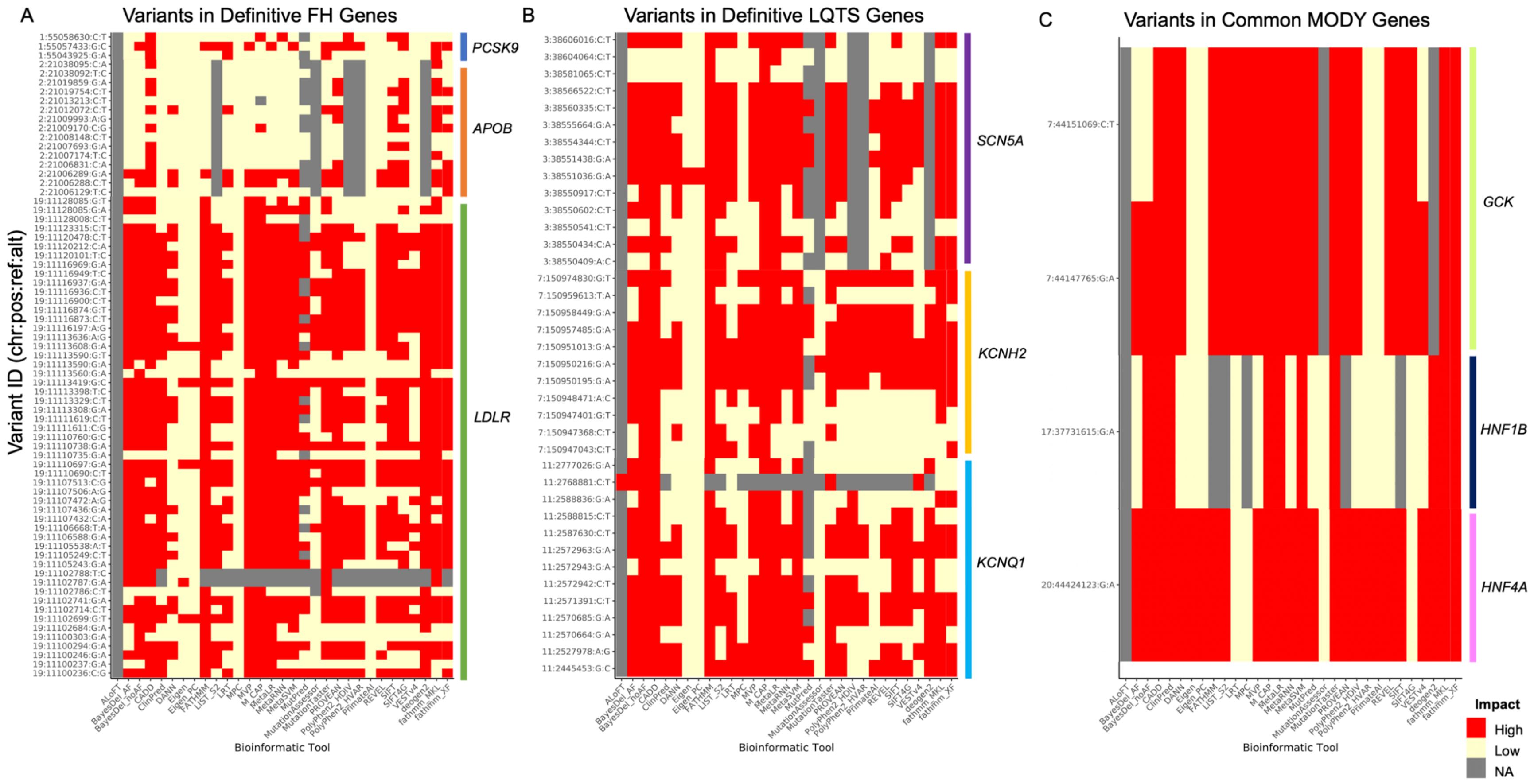

Supplementary Figure 8. Heatmap of bioinformatic tool functional predictions for variants with conflicting assertions with large effect sizes. Red boxes represent high predicted functional impact, yellow boxes represent low predicted functional impact, and grey boxes represent no prediction available. Panels A, B, and C display data for rare variants associated with LDL-C, QTc, and HbA1c endophenotypes, respectively. Definitive familial hypercholesterolemia (FH) genes include *LDLR, APOB, PCSK9*. Definitive long-QT syndrome (LQTS) genes include *KCNQ1, KCNH2, SCN5A*. Common maturity-onset diabetes of the young (MODY) genes include *HNF1A, HNF1B, HNF4A, GCK*; the color bar indicates the corresponding gene for the variants shown.

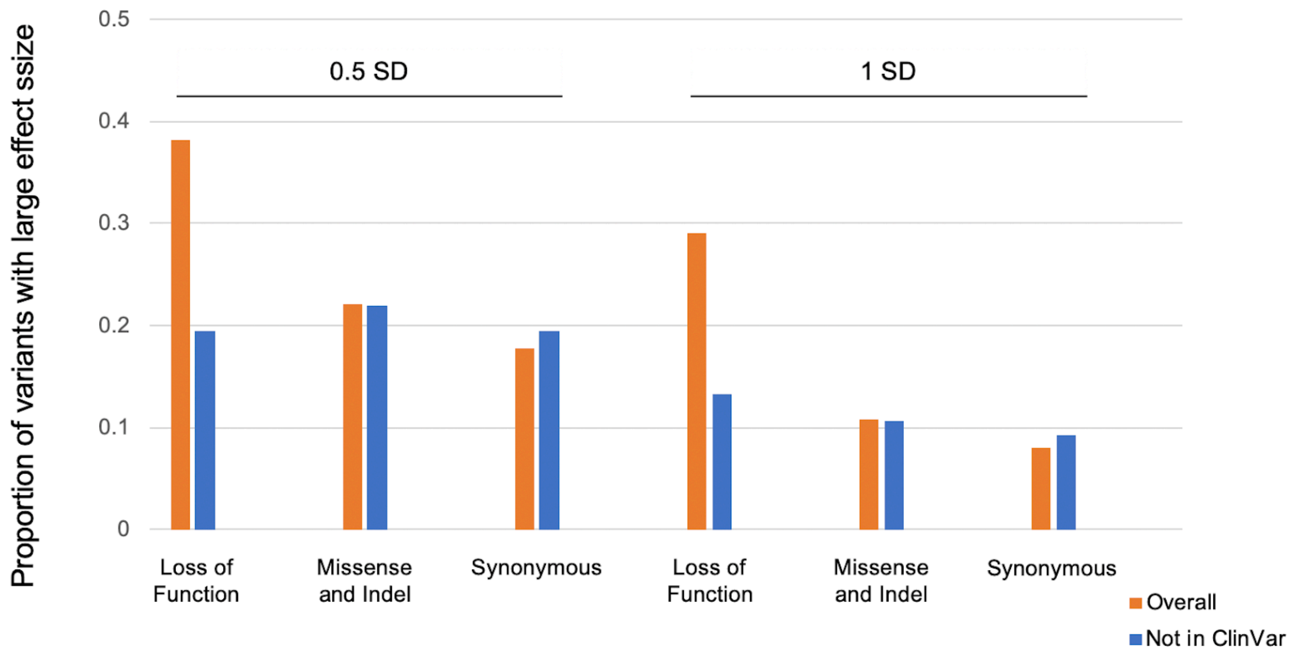

Supplementary Figure 9. Proportion of large effect size variants by variant consequence. Loss of function includes frameshift, stop-gained, and splice altering variants. Large effect size refers to variants with effect size > 0.5 standard deviations of the endophenotype distribution in the UK Biobank.

Supplementary Note 1

This work was supported by funding from: the National Institutes of Health (R01HL139731, R01HL157635) and American Heart Association (18SFRN34250007) to S.A.L., the Fondation Leducq (14CVD01), the National Institutes of Health (1RO1HL092577, K24HL105780) and American Heart Association (18SFRN34110082) to P.T.E., the National Institutes of Health (NHLBI K08HL153950) to N.A.M., the National Institutes of Health (1R01HL139731) and American Heart Association (18SFRN34110082) to L-C.W., the National Institutes of Health (NHLBI K08HL159346) to J.P.P., and the National Institutes of Health (R01HL092577) and American Heart Association (AF AHA_18SFRN34110082, 75N92019D00031) to E.J.B.

UK Biobank: Use of UK Biobank data was performed under application number 17488 and was approved by the local Massachusetts General Hospital institutional review board.

Trans-Omics in Precision Medicine (TOPMed) program: Whole genome sequencing for the Trans-Omics in Precision Medicine (TOPMed) program was supported by the National Heart, Lung and Blood Institute (NHLBI). Whole genome sequencing for "NHLBI TOPMed: the Atherosclerosis Risk in Communities" (phs001211.v1.p1) was performed at the Broad Institute of MIT and Harvard (3R01HL092577-06S1) and Baylor Human Genome Sequencing Center (3U54HG003273-12S2, HHSN268201500015C). Whole genome sequencing for "NHLBI TOPMed: Genetics of Cardiometabolic Health in the Amish" (phs000956.v1.p1) was performed at the Broad Institute of MIT and Harvard (3R01HL121007-01S1). Whole genome sequencing for "NHLBI TOPMed: Mount Sinai BioMe Biobank" (phs001644.v1.p1) was performed at the Baylor Human Genome Sequencing Center (HHSN268201600033I) and McDonnell genome institute (HHSN268201600037I). Whole genome sequencing for "NHLBI TOPMed: Cleveland Family Study - Whole genome sequencing Collaboration" (phs000954.v1.p1) was performed at the University of Washington northwest genomics center (3R01HL098433-05S1, HHSN268201600032I). Whole genome sequencing for "NHLBI TOPMed: Cardiovascular Health Study" (phs001368.v1.p1) was performed at the Baylor Human Genome Sequencing Center (3U54HG003273-12S2, HHSN268201500015C, HHSN268201600033I). Whole genome sequencing for "NHLBI TOPMed: Framingham Heart Study" (phs000974.v1.p1) was performed at the Broad institute of MIT and Harvard (3R01HL092577-06S1, 3U54HG003067-12S2). Whole genome sequencing for "NHLBI TOPMed: The Jackson Heart Study" (phs000964.v1.p1) was performed at the University of Washington Northwest Genomics Center (HHSN268201100037C). Whole genome sequencing for "NHLBI TOPMed: Multi-Ethnic Study of Atherosclerosis" (phs001416.v1.p1) was performed at the Broad Institute of MIT and Harvard (3U54HG003067-13S1). Whole genome sequencing for "NHLBI TOPMed: Womens Health Initiative" (phs001237.v1.p1) was performed at the Broad Institute of MIT and Harvard (HHSN268201500014C). Core support including read mapping and genotype calling, along with variant quality metrics and filtering were provided by the TOPMed Informatics Research Center (3R01HL-117626-02S1; contract HHSN268201800002I). Phenotype harmonization, data management, sample-identity QC, and general study coordination were provided by the TOPMed Data Coordinating Center (3R01HL-120393-02S1; contract HHSN268201800001I). We gratefully acknowledge the studies and participants who provided biological samples and data for TOPMed.

TOPMed Study Specific Acknowledgements:
Amish: Genetics of Cardiometabolic Health in the Amish

The TOPMed component of the Amish Research Program was supported by NIH grants R01 HL121007, U01 HL072515, and R01 AG18728. See publication: PMID: 18440328

BioMe: BioMe Biobank at Mount Sinai
The Mount Sinai BioMe Biobank has been supported by The Andrea and Charles Bronfman Philanthropies and in part by Federal funds from the NHLBI and NHGRI (U01HG00638001; U01HG007417; X01HL134588). We thank all participants in the Mount Sinai Biobank. We also thank all our recruiters who have assisted and continue to assist in data collection and management and are grateful for the computational resources and staff expertise provided by Scientific Computing at the Icahn School of Medicine at Mount Sinai.

ARIC: *Atherosclerosis Risk in Communities study*
The Atherosclerosis Risk in Communities study has been funded in whole or in part with Federal funds from the National Heart, Lung, and Blood Institute, National Institutes of Health, Department of Health and Human Services (contract numbers HHSN268201700001I, HHSN268201700002I, HHSN268201700003I, HHSN268201700004I and HHSN268201700005I). The authors thank the staff and participants of the ARIC study for their important contributions.

WGS for "NHLBI TOPMed: Atherosclerosis Risk in Communities (ARIC) Study" (phs001211) was performed at the Baylor College of Medicine Human Genome Sequencing Center (HHSN268201500015C and 3U54HG003273-12S2) and the Broad Institute for MIT and Harvard (3R01HL092577- 06S1). Centralized read mapping and genotype calling, along with variant quality metrics and filtering were provided by the TOPMed Informatics Research Center (3R01HL-117626-02S1). Phenotype harmonization, data management, sample-identity QC, and general study coordination, were provided by the TOPMed Data Coordinating Center (3R01HL-120393- 02S1). We gratefully acknowledge the studies and participants who provided biological samples and data for TOPMed.

CHS: Cardiovascular Health Study
Cardiovascular Health Study: This research was supported by contracts HHSN268201200036C, HHSN268200800007C, HHSN268201800001C, N01HC55222, N01HC85079, N01HC85080, N01HC85081, N01HC85082, N01HC85083, N01HC85086, 75N92021D00006 and grants U01HL080295 and U01HL130114 from the National Heart, Lung, and Blood Institute (NHLBI), with additional contribution from the National Institute of Neurological Disorders and Stroke (NINDS). Additional support was provided by R01AG023629 from the National Institute on Aging (NIA). A full list of principal CHS investigators and institutions can be found at CHS-NHLBI.org. The content is solely the responsibility of the authors and does not necessarily represent the official views of the National Institutes of Health.

CFS: Cleveland Family Study
The Cleveland Family Study has been supported in part by National Institutes of Health grants [R01-HL046380, KL2-RR024990, R35-HL135818, and R01-HL113338].

FHS: Framingham Heart Study
The Framingham Heart Study (FHS) acknowledges the support of contracts NO1-HC-25195, HHSN268201500001I and 75N92019D00031 from the National Heart, Lung and Blood Institute and grant supplement R01 HL092577-06S1 for this research. We also acknowledge the

dedication of the FHS study participants without whom this research would not be possible. Dr. Vasan is supported in part by the Evans Medical Foundation and the Jay and Louis Coffman Endowment from the Department of Medicine, Boston University School of Medicine.

JHS: Jackson Heart Study
The Jackson Heart Study (JHS) is supported and conducted in collaboration with Jackson State University (HHSN268201800013I), Tougaloo College (HHSN268201800014I), the Mississippi State Department of Health (HHSN268201800015I) and the University of Mississippi Medical Center (HHSN268201800010I, HHSN268201800011I and HHSN268201800012I) contracts from the National Heart, Lung, and Blood Institute (NHLBI) and the National Institute on Minority Health and Health Disparities (NIMHD). The authors also wish to thank the staffs and participants of the JHS.

The views expressed in this manuscript are those of the authors and do not necessarily represent the views of the National Heart, Lung, and Blood Institute; the National Institutes of Health; or the U.S. Department of Health and Human Services.

MESA: Multi-Ethnic Study of Atherosclerosis
MESA and the MESA SHARe project are conducted and supported by the National Heart, Lung, and Blood Institute (NHLBI) in collaboration with MESA investigators. Support for MESA is provided by contracts 75N92020D00001, HHSN268201500003I, N01-HC-95159, 75N92020D00005, N01-HC-95160, 75N92020D00002, N01-HC-95161, 75N92020D00003, N01-HC-95162, 75N92020D00006, N01-HC-95163, 75N92020D00004, N01-HC-95164, 75N92020D00007, N01-HC-95165, N01-HC-95166, N01-HC-95167, N01-HC-95168, N01-HC-95169, UL1-TR-000040, UL1-TR-001079, UL1-TR-001420. Funding for SHARe genotyping was provided by NHLBI Contract N02-HL-64278. Genotyping was performed at Affymetrix (Santa Clara, California, USA) and the Broad Institute of Harvard and MIT (Boston, Massachusetts, USA) using the Affymetrix Genome-Wide Human SNP Array 6.0. MESA Family is conducted and supported by the National Heart, Lung, and Blood Institute (NHLBI) in collaboration with MESA investigators. Support is provided by grants and contracts R01HL071051, R01HL071205, R01HL071250, R01HL071251, R01HL071258, R01HL071259, by the National Center for Research Resources, Grant UL1RR033176. The provision of genotyping data was supported in part by the National Center for Advancing Translational Sciences, CTSI grant UL1TR001881, and the National Institute of Diabetes and Digestive and Kidney Disease Diabetes Research Center (DRC) grant DK063491 to the Southern California Diabetes Endocrinology Research Center.

WHI: Women's Health Initiative
The WHI program is funded by the National Heart, Lung, and Blood Institute, National Institutes of Health, U.S. Department of Health and Human Services through contracts 75N92021D00001, 75N92021D00002, 75N92021D00003, 75N92021D00004, 75N92021D00005.

TOPMed Omics Support Acknowledgements:

TOPMed Accession Number	TOPMed Project	Parent Study	TOPMed Phase	Omics Center	Omics Support	Omics Type
phs000956	Amish	Amish	1	Broad Genomics	3R01HL121007-01S1	WGS
phs001211	AFGen	ARIC AFGen	1	Broad Genomics	3R01HL092577-06S1	WGS
phs001211	VTE	ARIC	2	Baylor	3U54HG003273-12S2 / HHSN268201500015C	WGS
phs001644	AFGen	BioMe AFGen	2.4	MGI	3UM1HG008853-01S2	WGS
phs001644	BioMe	BioMe	3	Baylor	HHSN268201600033I	WGS
phs001644	BioMe	BioMe	3	MGI	HHSN268201600037I	WGS
phs000954	CFS	CFS	1	NWGC	3R01HL098433-05S1	WGS
phs000954	CFS	CFS	3.5	NWGC	HHSN268201600032I	WGS
phs001368	CHS	CHS	3	Baylor	HHSN268201600033I	WGS
phs001368	VTE	CHS VTE	2	Baylor	3U54HG003273-12S2 / HHSN268201500015C	WGS
phs000974	AFGen	FHS AFGen	1	Broad Genomics	3R01HL092577-06S1	WGS
phs000974	FHS	FHS	1	Broad Genomics	3U54HG003067-12S2	WGS
phs000964	JHS	JHS	1	NWGC	HHSN268201100037C	WGS
phs001416	AA_CAC	MESA AA_CAC	2	Broad Genomics	HHSN268201500014C	WGS
phs001416	MESA	MESA	2	Broad Genomics	3U54HG003067-13S1	WGS
phs001237	WHI	WHI	2	Broad Genomics	HHSN268201500014C	WGS

ARIC: Atherosclerosis Risk in Communities (ARIC) study, Amish: Genetics of Cardiometabolic Health in the Amish, Baylor: Baylor College of Medicine Human Genome Sequencing Center, BioMe: Mount Sinai BioMe Biobank, Broad Genomics: Broad Institute Genomics Platform, CFS: Cleveland Family Study, CHS: Cardiovascular Health Study, FHS: Framingham Heart Study, JHS: Jackson Heart Study, MESA: Multi-Ethnic Study of Atherosclerosis, MGI: McDonnell Genome Institute, NWGC: Northwest Genomics Center, TOPMed: Transomics for Precision Medicine, WGS: Whole Genome Sequencing, WHI: Women's Health Initiative

Supplementary Note 2

Name	Institution(s)	Primary Department	Institution City	Institution State	Zip Code	Country
Abe, Namiko	New York Genome Center		New York	New York	10013	US
Abecasis, Gonçalo	University of Michigan		Ann Arbor	Michigan	48109	US
Albert, Christine	Brigham & Women's Hospital, Cedars Sinai		Boston	Massachusetts	02114	US
Almasy, Laura	Children's Hospital of Philadelphia, University of Pennsylvania		Philadelphia	Pennsylvania	19104	US
Alonso, Alvaro	Emory University	Epidemiology	Atlanta	Georgia	30322	US
Ament, Seth	University of Maryland		Baltimore	Maryland	21201	US
Anderson, Peter	University of Washington		Seattle	Washington	98195	US
Anugu, Pramod	University of Mississippi		Jackson	Mississippi	38677	US
Applebaum-Bowden, Deborah	National Institutes of Health		Bethesda	Maryland	20892	US
Arking, Dan	Johns Hopkins University		Baltimore	Maryland	21218	US
Arnett, Donna K	University of Kentucky		Lexington	Kentucky	40506	US
Ashley-Koch, Allison	Duke University		Durham	North Carolina	27708	US
Aslibekyan, Stella	University of Alabama		Birmingham	Alabama	35487	US
Assimes, Tim	Stanford University		Stanford	California	94305	US
Auer, Paul	University of Wisconsin Milwaukee		Milwaukee	Wisconsin	53211	US
Avramopoulos, Dimitrios	Johns Hopkins University		Baltimore	Maryland	21218	US
Barnard, John	Cleveland Clinic		Cleveland	Ohio	44195	US
Barnes, Kathleen	University of Colorado at Denver		Denver	Colorado	80204	US
Barr, R. Graham	Columbia University		New York	New York	10027	US
Barron-Casella, Emily	Johns Hopkins University		Baltimore	Maryland	21218	US
Beaty, Terri	Johns Hopkins University		Baltimore	Maryland	21218	US
Becker, Diane	Johns Hopkins University	Medicine	Baltimore	Maryland	21218	US
Becker, Lewis	Johns Hopkins University		Baltimore	Maryland	21218	US
Beer, Rebecca	National Heart, Lung, and Blood Institute, National Institutes of Health		Bethesda	Maryland	20892	US
Begum, Ferdouse	Johns Hopkins University		Baltimore	Maryland	21218	US
Beitelshees, Amber	University of Maryland		Baltimore	Maryland	21201	US
Benjamin, Emelia J.	Boston University	Boston University School of Medicine	Boston	Massachusetts	02118	US
Bezerra, Marcos	Fundação de Hematologia e Hemoterapia de Pernambuco - Hemope		Recife		52011 -000	BR
Bielak, Larry	University of Michigan		Ann Arbor	Michigan	48109	US
Bis, Joshua	University of Washington		Seattle	Washington	98195	US
Blackwell, Thomas	University of Michigan		Ann Arbor	Michigan	48109	US
Blangero, John	University of Texas Rio Grande Valley School of Medicine	Human Genetics	Brownsville	Texas	78520	US
Boerwinkle, Eric	University of Texas Health at Houston		Houston	Texas	77225	US
Bowden, Donald W.	Wake Forest Baptist Health	Department of Biochemistry	Winston-Salem	North Carolina	27157	US

Bowler, Russell	National Jewish Health	National Jewish Health	Denver	Colorado	80206	US
Brody, Jennifer	University of Washington		Seattle	Washington	98195	US
Broeckel, Ulrich	Medical College of Wisconsin		Milwaukee	Wisconsin	53226	US
Broome, Jai	University of Washington		Seattle	Washington	98195	US
Bunting, Karen	New York Genome Center		New York	New York	10013	US
Burchard, Esteban	University of California, San Francisco		San Francisco	California	94143	US
Buth, Erin	University of Washington	Biostatistics	Seattle	Washington	98195	US
Cade, Brian	Brigham & Women's Hospital	Brigham and Women's Hospital	Boston	Massachusetts	02115	US
Cardwell, Jonathan	University of Colorado at Denver		Denver	Colorado	80204	US
Carty, Cara	Women's Health Initiative		Seattle	Washington	98109	US
Casaburi, Richard	University of California, Los Angeles		Los Angeles	California	90095	US
Casella, James	Johns Hopkins University		Baltimore	Maryland	21218	US
Chaffin, Mark	Broad Institute		Cambridge	Massachusetts	02142	US
Chang, Christy	University of Maryland		Baltimore	Maryland	21201	US
Chasman, Daniel	Brigham & Women's Hospital	Division of Preventive Medicine	Boston	Massachusetts	02215	US
Chavan, Sameer	University of Colorado at Denver		Denver	Colorado	80204	US
Chen, Bo-Juen	New York Genome Center		New York	New York	10013	US
Chen, Wei-Min	University of Virginia		Charlottesville	Virginia	22903	US
Chen, Yii-Der Ida	Lundquist Institute		Charlottesville	Virginia	90502	US
Cho, Michael	Brigham & Women's Hospital		Boston	Massachusetts	02115	US
Choi, Seung Hoan	Broad Institute		Cambridge	Massachusetts	02142	US
Chuang, Lee-Ming	National Taiwan University	National Taiwan University Hospital	Taipei		10617	TW
Chung, Mina	Cleveland Clinic	Cleveland Clinic	Cleveland	Ohio	44195	US
Conomos, Matthew P.	University of Washington	Biostatistics	Seattle	Washington	98115	US
Cornell, Elaine	University of Vermont		Burlington	Vermont	05405	US
Correa, Adolfo	University of Mississippi	Medicine	Jackson	Mississippi	39216	US
Crandall, Carolyn	University of California, Los Angeles		Los Angeles	California	90095	US
Crapo, James	National Jewish Health		Denver	Colorado	80206	US
Cupples, L. Adrienne	Boston University	Biostatistics	Boston	Massachusetts	02118	US
Curran, Joanne	University of Texas Rio Grande Valley School of Medicine		Brownsville	Texas	78520	US
Curtis, Jeffrey	University of Michigan		Ann Arbor	Michigan	48109	US
Custer, Brian	Vitalant Research Institute		San Francisco	California	94118	US
Damcott, Coleen	University of Maryland		Baltimore	Maryland	21201	US
Darbar, Dawood	University of Illinois at Chicago		Chicago	Illinois	60607	US
Das, Sayantan	University of Michigan		Ann Arbor	Michigan	48109	US
David, Sean	University of Chicago			Illinois		US
Davis, Colleen	University of Washington		Seattle	Washington	98195	US
Daya, Michelle	University of Colorado at Denver		Denver	Colorado	80204	US
de Andrade, Mariza	Mayo Clinic	Health Sciences Research	Rochester	Minnesota	55905	US
DeBaun, Michael	Vanderbilt University		Nashville	Tennessee	37235	US
Deka, Ranjan	University of Cincinnati		Cincinnati	Ohio	45220	US
DeMeo, Dawn	Brigham & Women's Hospital		Boston	Massachusetts	02115	US
Devine, Scott	University of Maryland		Baltimore	Maryland	21201	US

Do, Ron	Icahn School of Medicine at Mount Sinai		New York	New York	10029	US
Duan, Qing	University of North Carolina		Chapel Hill	North Carolina	27599	US
Duggirala, Ravi	University of Texas Rio Grande Valley School of Medicine		Edinburg	Texas	78539	US
Durda, Jon Peter	University of Vermont		Burlington	Vermont	05405	US
Dutcher, Susan	Washington University in St Louis		St Louis	Missouri	63130	US
Eaton, Charles	Brown University		Providence	Rhode Island	02912	US
Ekunwe, Lynette	University of Mississippi		Jackson	Mississippi	38677	US
Ellinor, Patrick	Massachusetts General Hospital	Cardiovascular Research Center	Boston	Massachusetts	02114	US
Emery, Leslie	University of Washington		Seattle	Washington	98195	US
Farber, Charles	University of Virginia		Charlottesville	Virginia	22903	US
Farnam, Leanna	Brigham & Women's Hospital		Boston	Massachusetts	02115	US
Fingerlin, Tasha	National Jewish Health	Center for Genes, Environment and Health	Denver	Colorado	80206	US
Flickinger, Matthew	University of Michigan		Ann Arbor	Michigan	48109	US
Fornage, Myriam	University of Texas Health at Houston		Houston	Texas	77225	US
Franceschini, Nora	University of North Carolina	Epidemiology	Chapel Hill	North Carolina	27599	US
Fu, Mao	University of Maryland		Baltimore	Maryland	21201	US
Fullerton, Stephanie M.	University of Washington		Seattle	Washington	98195	US
Fulton, Lucinda	Washington University in St Louis		St Louis	Missouri	63130	US
Gabriel, Stacey	Broad Institute		Cambridge	Massachusetts	02142	US
Gan, Weiniu	National Heart, Lung, and Blood Institute, National Institutes of Health		Bethesda	Maryland	20892	US
Gao, Yan	University of Mississippi		Jackson	Mississippi	38677	US
Gass, Margery	Fred Hutchinson Cancer Research Center		Seattle	Washington	98109	US
Gelb, Bruce	Icahn School of Medicine at Mount Sinai		New York	New York	10029	US
Geng, Xiaoqi (Priscilla)	University of Michigan		Ann Arbor	Michigan	48109	US
Germer, Soren	New York Genome Center		New York	New York	10013	US
Gignoux, Chris	Stanford University		Stanford	California	94305	US
Gladwin, Mark	University of Pittsburgh		Pittsburgh	Pennsylvania	15260	US
Glahn, David	Yale University		New Haven	Connecticut	06520	US
Gogarten, Stephanie	University of Washington		Seattle	Washington	98195	US
Gong, Da-Wei	University of Maryland		Baltimore	Maryland	21201	US
Goring, Harald	University of Texas Rio Grande Valley School of Medicine		San Antonio	Texas	78229	US
Gu, C. Charles	Washington University in St Louis		St Louis	Missouri	63130	US
Guan, Yue	University of Maryland		Baltimore	Maryland	21201	US
Guo, Xiuqing	Lundquist Institute		Los Angeles	California	90502	US
Haessler, Jeff	Fred Hutchinson Cancer Research Center, Women's Health Initiative		Seattle	Washington	98109	US
Hall, Michael	University of Mississippi		Jackson	Mississippi	38677	US
Harris, Daniel	University of Maryland		Baltimore	Maryland	21201	US
Hawley, Nicola	Yale University		New Haven	Connecticut	06520	US
He, Jiang	Tulane University		New Orleans	Louisiana	70118	US
Heavner, Ben	University of Washington	Biostatistics	Seattle	Washington	98195	US

Heckbert, Susan	University of Washington		Seattle	Washington	98195	US
Hernandez, Ryan	McGill University, University of California, San Francisco					CA
Herrington, David	Wake Forest Baptist Health		Winston-Salem	North Carolina	27157	US
Hersh, Craig	Brigham & Women's Hospital	Channing Division of Network Medicine	Boston	Massachusetts	02115	US
Hidalgo, Bertha	University of Alabama		Birmingham	Alabama	35487	US
Hixson, James	University of Texas Health at Houston		Houston	Texas	77225	US
Hokanson, John	University of Colorado at Denver		Denver	Colorado	80204	US
Hong, Elliott	University of Maryland		Baltimore	Maryland	21201	US
Hoth, Karin	University of Iowa		Iowa City	Iowa	52242	US
Hsiung, Chao (Agnes)	National Health Research Institute Taiwan	Institute of Population Health Sciences, NHRI	Miaoli County		350	TW
Huston, Haley	Blood Works Northwest		Seattle	Washington	98105	US
Hwu, Chii Min	Taichung Veterans General Hospital Taiwan		Taichung City		407	TW
Irvin, Marguerite Ryan	University of Alabama		Birmingham	Alabama	35487	US
Jackson, Rebecca	Ohio State University Wexner Medical Center	Internal Medicine, DIvision of Endocrinology, Diabetes and Metabolism	Columbus	Ohio	43210	US
Jain, Deepti	University of Washington		Seattle	Washington	98195	US
Jaquish, Cashell	National Heart, Lung, and Blood Institute, National Institutes of Health		Bethesda	Maryland	20892	US
Jhun, Min A	University of Michigan		Ann Arbor	Michigan	48109	US
Johnsen, Jill	Blood Works Northwest, University of Washington		Seattle	Washington	98102	US
Johnson, Andrew	National Heart, Lung, and Blood Institute, National Institutes of Health		Bethesda	Maryland	20892	US
Johnson, Craig	University of Washington		Seattle	Washington	98195	US
Johnston, Rich	Emory University		Atlanta	Georgia	30322	US
Jones, Kimberly	Johns Hopkins University		Baltimore	Maryland	21218	US
Kang, Hyun Min	University of Michigan	Biostatistics	Ann Arbor	Michigan	48109	US
Kaplan, Robert	Albert Einstein College of Medicine		New York	New York	10461	US
Kardia, Sharon	University of Michigan		Ann Arbor	Michigan	48109	US
Kathiresan, Sekar	Broad Institute		Cambridge	Massachusetts	02142	US
Kaufman, Laura	Brigham & Women's Hospital		Boston	Massachusetts	02115	US
Kelly, Shannon	Vitalant Research Institute		San Francisco	California	94118	US
Kenny, Eimear	Icahn School of Medicine at Mount Sinai		New York	New York	10029	US
Kessler, Michael	University of Maryland		Baltimore	Maryland	21201	US
Khan, Alyna	University of Washington		Seattle	Washington	98195	US
Kinney, Greg	University of Colorado at Denver		Denver	Colorado	80204	US
Konkle, Barbara	Blood Works Northwest		Seattle	Washington	98104	US
Kooperberg, Charles	Fred Hutchinson Cancer Research Center		Seattle	Washington	98109	US

Kramer, Holly	Loyola University	Public Health Sciences	Maywood	Illinois	60153	US
Krauter, Stephanie	University of Washington		Seattle	Washington	98195	US
Lange, Christoph	Harvard School of Public Health	Biostats	Boston	Massachusetts	02115	US
Lange, Ethan	University of Colorado at Denver		Denver	Colorado	80204	US
Lange, Leslie	University of Colorado at Denver		Denver	Colorado	80204	US
Laurie, Cathy	University of Washington		Seattle	Washington	98195	US
Laurie, Cecelia	University of Washington		Seattle	Washington	98195	US
LeBoff, Meryl	Brigham & Women's Hospital		Boston	Massachusetts	02115	US
Lee, Jiwon	Brigham & Women's Hospital					
Lee, Seunggeun Shawn	University of Michigan		Ann Arbor	Michigan	48109	US
Lee, Wen-Jane	Taichung Veterans General Hospital Taiwan		Taichung City		407	TW
LeFaive, Jonathon	University of Michigan		Ann Arbor	Michigan	48109	US
Levine, David	University of Washington		Seattle	Washington	98195	US
Levy, Dan	National Heart, Lung, and Blood Institute, National Institutes of Health		Bethesda	Maryland	20892	US
Lewis, Joshua	University of Maryland		Baltimore	Maryland	21201	US
Li, Yun	University of North Carolina		Chapel Hill	North Carolina	27599	US
Lin, Honghuang	Boston University		Boston	Massachusetts	02215	US
Lin, Keng Han	University of Michigan		Ann Arbor	Michigan	48109	US
Lin, Xihong	Harvard School of Public Health					
Liu, Simin	Brown University, Women's Health Initiative	Epidemiology	Providence	Rhode Island	02912	US
Liu, Yongmei	Duke University	Cardiology	Durham	North Carolina	27701	US
Loos, Ruth J.F.	Icahn School of Medicine at Mount Sinai	The Charles Bronfman Institute for Personalized Medicine	New York	New York	10029	US
Lubitz, Steven	Massachusetts General Hospital		Boston	Massachusetts	02114	US
Lunetta, Kathryn	Boston University		Boston	Massachusetts	02215	US
Luo, James	National Heart, Lung, and Blood Institute, National Institutes of Health		Bethesda	Maryland	20892	US
Mahaney, Michael	University of Texas Rio Grande Valley School of Medicine		Brownsville	Texas	78520	US
Make, Barry	Johns Hopkins University		Baltimore	Maryland	21218	US
Manichaikul, Ani	University of Virginia		Charlottesville	Virginia	22903	US
Manson, JoAnn	Brigham & Women's Hospital		Boston	Massachusetts	02115	US
Margolin, Lauren	Broad Institute		Cambridge	Massachusetts	02142	US
Martin, Lisa	George Washington University		Washington	District of Columbia	20052	US
Mathai, Susan	University of Colorado at Denver		Denver	Colorado	80204	US
Mathias, Rasika	Johns Hopkins University		Baltimore	Maryland	21218	US
McArdle, Patrick	University of Maryland		Baltimore	Maryland	21201	US
McDonald, Merry-Lynn	University of Alabama		Birmingham	Alabama	35487	US
McFarland, Sean	Harvard University		Cambridge	Massachusetts	02138	US
McGarvey, Stephen	Brown University		Providence	Rhode Island	02912	US
McHugh, Caitlin	University of Washington	Biostatistics	Seattle	Washington	98145	US
Mei, Hao	University of Mississippi		Jackson	Mississippi	38677	US
Meyers, Deborah A	University of Arizona		Tucson	Arizona	85721	US

Mikulla, Julie	National Heart, Lung, and Blood Institute, National Institutes of Health		Bethesda	Maryland	20892	US
Min, Nancy	University of Mississippi		Jackson	Mississippi	38677	US
Minear, Mollie	National Heart, Lung, and Blood Institute, National Institutes of Health		Bethesda	Maryland	20892	US
Minster, Ryan L	University of Pittsburgh		Pittsburgh	Pennsylvania	15260	US
Mitchell, Braxton D.	University of Maryland		Baltimore	Maryland	21201	US
Montasser, May E.	University of Maryland		Baltimore	Maryland	21201	US
Musani, Solomon	University of Mississippi	Medicine	Jackson	Mississippi	39213	US
Mwasongwe, Stanford	University of Mississippi		Jackson	Mississippi	38677	US
Mychaleckyj, Josyf C	University of Virginia		Charlottesville	Virginia	22903	US
Nadkarni, Girish	Icahn School of Medicine at Mount Sinai		New York	New York	10029	US
Naik, Rakhi	Johns Hopkins University		Baltimore	Maryland	21218	US
Naseri, Take	Ministry of Health, Government of Samoa		Apia			WS
Natarajan, Pradeep	Broad Institute, Harvard University, Massachusetts General Hospital		Cambridge	Massachusetts	02138	US
Nekhai, Sergei	Howard University		Washington	District of Columbia	20059	US
Nelson, Sarah C.	University of Washington	Biostatistics	Seattle	Washington	98195	US
Nickerson, Deborah	University of Washington		Seattle	Washington	98195	US
North, Kari	University of North Carolina		Chapel Hill	North Carolina	27599	US
O'Connell, Jeff	University of Maryland		Balitmore	Maryland	21201	US
O'Connor, Tim	University of Maryland		Baltimore	Maryland	21201	US
Ochs-Balcom, Heather	University at Buffalo		Buffalo	New York	14260	US
Palmer, Nicholette	Wake Forest Baptist Health	Biochemistry	Winston-Salem	North Carolina	27157	US
Pankow, James	University of Minnesota		Minneapolis	Minnesota	55455	US
Papanicolaou, George	National Heart, Lung, and Blood Institute, National Institutes of Health		Bethesda	Maryland	20892	US
Parker, Margaret	Brigham & Women's Hospital		Boston	Massachusetts	02115	US
Parsa, Afshin	University of Maryland		Baltimore	Maryland	21201	US
Penchev, Sara	National Jewish Health		Denver	Colorado	80206	US
Peralta, Juan Manuel	University of Texas Rio Grande Valley School of Medicine		Edinburg	Texas	78539	US
Perez, Marco	Stanford University		Stanford	California	94305	US
Perry, James	University of Maryland		Baltimore	Maryland	21201	US
Peters, Ulrike	Fred Hutchinson Cancer Research Center, University of Washington		Seattle	Washington	98109	US
Peyser, Patricia	University of Michigan		Ann Arbor	Michigan	48109	US
Phillips, Lawrence S	Emory University		Atlanta	Georgia	30322	US
Phillips, Sam	University of Washington		Seattle	Washington	98195	US
Pollin, Toni	University of Maryland		Baltimore	Maryland	21201	US
Post, Wendy	Johns Hopkins University	Cardiology/Medicine	Baltimore	Maryland	21218	US
Powers Becker, Julia	University of Colorado at Denver	Medicine	Denver	Colorado	80204	US
Preethi Boorgula, Meher	University of Colorado at Denver		Denver	Colorado	80204	US

Preuss, Michael	Icahn School of Medicine at Mount Sinai		New York	New York	10029	US
Prokopenko, Dmitry	Harvard University		Cambridge	Massachusetts	02138	US
Psaty, Bruce	University of Washington		Seattle	Washington	98195	US
Qasba, Pankaj	National Heart, Lung, and Blood Institute, National Institutes of Health		Bethesda	Maryland	20892	US
Qiao, Dandi	Brigham & Women's Hospital		Boston	Massachusetts	02115	US
Qin, Zhaohui	Emory University		Atlanta	Georgia	30322	US
Rafaels, Nicholas	University of Colorado at Denver		Denver	Colorado	80045	US
Raffield, Laura	University of North Carolina	Genetics	Chapel Hill	North Carolina	27599	US
Rao, D.C.	Washington University in St Louis		St Louis	Missouri	63130	US
Rasmussen-Torvik, Laura	Northwestern University		Chicago	Illinois	60208	US
Ratan, Aakrosh	University of Virginia		Charlottesville	Virginia	22903	US
Redline, Susan	Brigham & Women's Hospital		Boston	Massachusetts	02115	US
Reed, Robert	University of Maryland		Baltimore	Maryland	21201	US
Regan, Elizabeth	National Jewish Health		Denver	Colorado	80206	US
Reiner, Alex	Fred Hutchinson Cancer Research Center, University of Washington		Seattle	Washington	98109	US
Reupena, Muagututi'a Sefuiva	Lutia I Puava Ae Mapu I Fagalele		Apia			WS
Rice, Ken	University of Washington		Seattle	Washington	98195	US
Rich, Stephen	University of Virginia		Charlottesville	Virginia	22903	US
Roden, Dan	Vanderbilt University	Medicine, Pharmacology, Biomedicla Informatics	Nashville	Tennessee	37235	US
Roselli, Carolina	Broad Institute		Cambridge	Massachusetts	02142	US
Rotter, Jerome	Lundquist Institute		Los Angeles	California	90502	US
Ruczinski, Ingo	Johns Hopkins University		Baltimore	Maryland	21218	US
Russell, Pamela	University of Colorado at Denver		Denver	Colorado	80204	US
Ruuska, Sarah	Blood Works Northwest		Seattle	Washington	98107	US
Ryan, Kathleen	University of Maryland		Baltimore	Maryland	21201	US
Sabino, Ester Cerdeira	Universidade de Sao Paulo	Faculdade de Medicina	Sao Paulo		01310 000	BR
Sakornsakolpat, Phuwanat	Brigham & Women's Hospital		Boston	Massachusetts	02115	US
Salimi, Shabnam	University of Maryland		Baltimore	Maryland	21201	US
Salzberg, Steven	Johns Hopkins University		Baltimore	Maryland	21218	US
Sandow, Kevin	Lundquist Institute	TGPS	Torrance	California	90502	US
Sankaran, Vijay G.	Broad Institute, Harvard University	Division of Hematology/Oncology	Boston	Massachusetts	02115	US
Scheller, Christopher	University of Michigan		Ann Arbor	Michigan	48109	US
Schmidt, Ellen	University of Michigan		Ann Arbor	Michigan	48109	US
Schwander, Karen	Washington University in St Louis		St Louis	Missouri	63130	US
Schwartz, David	University of Colorado at Denver		Denver	Colorado	80204	US
Sciurba, Frank	University of Pittsburgh		Pittsburgh	Pennsylvania	15260	US
Seidman, Christine	Harvard Medical School	Genetics	Boston	Massachusetts	02115	US
Seidman, Jonathan	Harvard Medical School		Boston	Massachusetts	02115	US

Sheehan, Vivien	Baylor College of Medicine	Pediatrics	Houston	Texas	77030	US
Shetty, Amol	University of Maryland		Baltimore	Maryland	21201	US
Shetty, Aniket	University of Colorado at Denver		Denver	Colorado	80204	US
Sheu, Wayne Hui-Heng	Taichung Veterans General Hospital Taiwan		Taichung City		407	TW
Shoemaker, M. Benjamin	Vanderbilt University	Medicine/Cardiology	Nashville	Tennessee	37212	US
Silver, Brian	UMass Memorial Medical Center		Worcester	Massachusetts	01655	US
Silverman, Edwin	Brigham & Women's Hospital		Boston	Massachusetts	02115	US
Smith, Jennifer	University of Michigan		Ann Arbor	Michigan	48109	US
Smith, Josh	University of Washington		Seattle	Washington	98195	US
Smith, Nicholas	University of Washington	Epidemiology	Seattle	Washington	98195	US
Smith, Tanja	New York Genome Center		New York	New York	10013	US
Smoller, Sylvia	Albert Einstein College of Medicine		New York	New York	10461	US
Snively, Beverly	Wake Forest Baptist Health	Biostatistical Sciences	Winston-Salem	North Carolina	27157	US
Sofer, Tamar	Brigham & Women's Hospital		Boston	Massachusetts	02115	US
Sotoodehnia, Nona	University of Washington		Seattle	Washington	98195	US
Stilp, Adrienne	University of Washington		Seattle	Washington	98195	US
Streeten, Elizabeth	University of Maryland		Baltimore	Maryland	21201	US
Su, Jessica Lasky	Brigham & Women's Hospital					
Sung, Yun Ju	Washington University in St Louis		St Louis	Missouri	63130	US
Sylvia, Jody	Brigham & Women's Hospital		Boston	Massachusetts	02115	US
Szpiro, Adam	University of Washington		Seattle	Washington	98195	US
Sztalryd, Carole	University of Maryland		Baltimore	Maryland	21201	US
Taliun, Daniel	University of Michigan		Ann Arbor	Michigan	48109	US
Tang, Hua	Stanford University	Genetics	Stanford	California	94305	US
Taub, Margaret	Johns Hopkins University		Baltimore	Maryland	21218	US
Taylor, Kent D.	Lundquist Institute	Institute for Translational Genomics and Populations Sciences	Torrance	California	90502	US
Taylor, Simeon	University of Maryland		Baltimore	Maryland	21201	US
Telen, Marilyn	Duke University		Durham	North Carolina	27708	US
Thornton, Timothy A.	University of Washington		Seattle	Washington	98195	US
Tinker, Lesley	Women's Health Initiative		Seattle	Washington	98109	US
Tirschwell, David	University of Washington		Seattle	Washington	98195	US
Tiwari, Hemant	University of Alabama		Birmingham	Alabama	35487	US
Tracy, Russell	University of Vermont	Pathology & Laboratory Medicine	Burlington	Vermont	05405	US
Tsai, Michael	University of Minnesota		Minneapolis	Minnesota	55455	US
Vaidya, Dhananjay	Johns Hopkins University		Baltimore	Maryland	21218	US
VandeHaar, Peter	University of Michigan		Ann Arbor	Michigan	48109	US
Vasan, Ramachandran S.	Boston University		Boston	Massachusetts	02215	US
Vrieze, Scott	University of Colorado at Boulder, University of Minnesota		Boulder	Colorado	80309	US
Walker, Tarik	University of Colorado at Denver		Denver	Colorado	80204	US
Wallace, Robert	University of Iowa		Iowa City	Iowa	52242	US
Walts, Avram	University of Colorado at Denver		Denver	Colorado	80204	US

Wan, Emily	Brigham & Women's Hospital		Boston	Massachusetts	02115	US
Wang, Fei Fei	University of Washington		Seattle	Washington	98195	US
Wang, Heming	Brigham & Women's Hospital, Partners.org					
Watson, Karol	University of California, Los Angeles		Los Angeles	California	90095	US
Weeks, Daniel E.	University of Pittsburgh		Pittsburgh	Pennsylvania	15260	US
Weir, Bruce	University of Washington		Seattle	Washington	98195	US
Weiss, Scott	Brigham & Women's Hospital		Boston	Massachusetts	02115	US
Weng, Lu-Chen	Massachusetts General Hospital		Boston	Massachusetts	02114	US
Willer, Cristen	University of Michigan	Internal Medicine	Ann Arbor	Michigan	48109	US
Williams, Kayleen	University of Washington		Seattle	Washington	98195	US
Williams, L. Keoki	Henry Ford Health System		Detroit	Michigan	48202	US
Wilson, Carla	Brigham & Women's Hospital		Boston	Massachusetts	02115	US
Wilson, James	Beth Israel Deaconess Medical Center	Cardiology				US
Wong, Quenna	University of Washington		Seattle	Washington	98195	US
Xu, Huichun	University of Maryland		Baltimore	Maryland	21201	US
Yanek, Lisa	Johns Hopkins University		Baltimore	Maryland	21218	US
Yang, Ivana	University of Colorado at Denver		Denver	Colorado	80204	US
Yang, Rongze	University of Maryland		Baltimore	Maryland	21201	US
Zaghloul, Norann	University of Maryland		Baltimore	Maryland	21201	US
Zekavat, Maryam	Broad Institute		Cambridge	Massachusetts	02142	US
Zhang, Yingze	University of Pittsburgh	Medicine	Pittsburgh	Pennsylvania	15260	US
Zhao, Snow Xueyan	National Jewish Health		Denver	Colorado	80206	US
Zhao, Wei	University of Michigan		Ann Arbor	Michigan	48109	US
Zhi, Degui	University of Texas Health at Houston		Houston	Texas	77225	US
Zhou, Xiang	University of Michigan		Ann Arbor	Michigan	48109	US
Zhu, Xiaofeng	Case Western Reserve University	Department of Population and Quantitative Health Sciences	Cleveland	Ohio	44106	US
Zody, Michael	New York Genome Center		New York	New York	10013	US
Zoellner, Sebastian	University of Michigan		Ann Arbor	Michigan	48109	US

Supplementary References

1. Chang, C. C. *et al.* Second-generation PLINK: rising to the challenge of larger and richer datasets. *Gigascience* **4**, 7 (2015).
2. Alexander, D. H., Novembre, J. & Lange, K. Fast model-based estimation of ancestry in unrelated individuals. *Genome Res.* **19**, 1655–1664 (2009).
3. UKB : Data-Field 42001. https://biobank.ndph.ox.ac.uk/showcase/field.cgi?id=42001.
