# Supplementary material for: Endophenotype effect sizes support variant pathogenicity in monogenic disease susceptibility genes
Source: Nat Commun. 2022 Aug 30;13:5106. doi: 10.1038/s41467-022-32009-5 (PMC9427940; doi:10.1038/s41467-022-32009-5)
Supplement: Supplementary file 3 — Description of Additional Supplementary Files [file 41467_2022_32009_MOESM3_ESM.pdf]

File name: Supplementary Data 1

Description: Characteristics of variants of uncertain significance and conflicting assertions with large effect sizes

File name: Supplementary Data 2

Description: Characteristics of variants not reported in ClinVar with large effect sizes
