# Supplementary material for: Endophenotype effect sizes support variant pathogenicity in monogenic disease susceptibility genes
Source: Nat Commun. 2022 Aug 30;13:5106. doi: 10.1038/s41467-022-32009-5 (PMC9427940; doi:10.1038/s41467-022-32009-5)
Supplement: Supplementary file 5 — Reporting Summary [file 41467_2022_32009_MOESM5_ESM.pdf]

nature portfolio

Corresponding author(s): Steven Lubitz

Last updated by author(s): 06/06/2022

Reporting Summary

Nature Portfolio wishes to improve the reproducibility of the work that we publish. This form provides structure for consistency and transparency in reporting. For further information on Nature Portfolio policies, see our Editorial Policies and the Editorial Policy Checklist.

Statistics

For all statistical analyses, confirm that the following items are present in the figure legend, table legend, main text, or Methods section.

n/a	Confirmed	
☐	☒	The exact sample size (*n*) for each experimental group/condition, given as a discrete number and unit of measurement
☐	☒	A statement on whether measurements were taken from distinct samples or whether the same sample was measured repeatedly
☐	☒	The statistical test(s) used AND whether they are one- or two-sided<br>*Only common tests should be described solely by name; describe more complex techniques in the Methods section.*
☐	☒	A description of all covariates tested
☐	☒	A description of any assumptions or corrections, such as tests of normality and adjustment for multiple comparisons
☐	☒	A full description of the statistical parameters including central tendency (e.g. means) or other basic estimates (e.g. regression coefficient) AND variation (e.g. standard deviation) or associated estimates of uncertainty (e.g. confidence intervals)
☐	☒	For null hypothesis testing, the test statistic (e.g. *F*, *t*, *r*) with confidence intervals, effect sizes, degrees of freedom and *P* value noted<br>*Give P values as exact values whenever suitable.*
☒	☐	For Bayesian analysis, information on the choice of priors and Markov chain Monte Carlo settings
☒	☐	For hierarchical and complex designs, identification of the appropriate level for tests and full reporting of outcomes
☒	☐	Estimates of effect sizes (e.g. Cohen's *d*, Pearson's *r*), indicating how they were calculated

Our web collection on statistics for biologists contains articles on many of the points above.

Software and code

Policy information about availability of computer code

Data collection	We did not use specific software to collect data.
Data analysis	KING2, ADMIXTURE version 1.3.0, PLINK 2.0, PCAir, GENESIS version 2.14.3, R version 3.6 (including pROC, ggplot2 packages), dbNSFP v4.2a, SpliceAI

For manuscripts utilizing custom algorithms or software that are central to the research but not yet described in published literature, software must be made available to editors and reviewers. We strongly encourage code deposition in a community repository (e.g. GitHub). See the Nature Portfolio guidelines for submitting code & software for further information.

Data

Policy information about availability of data

All manuscripts must include a data availability statement. This statement should provide the following information, where applicable:

- Accession codes, unique identifiers, or web links for publicly available datasets
- A description of any restrictions on data availability
- For clinical datasets or third party data, please ensure that the statement adheres to our policy

To facilitate use of our results into potential clinical practice and research, we will make variant level association results from the present analysis publicly accessible in the Cardiovascular Disease Knowledge Portal ((https://cvd.hugeamp.org/downloads.html). Access to individual-level UK Biobank data is available to researchers through application on the UK Biobank website (https://www.ukbiobank.ac.uk). The use of UK Biobank data was performed under application number 17488. Data availability from TOPMed and FOURIER are subject to controlled access. Other datasets used in this manuscript include: the ClinVar database (https://www.ncbi.nlm.nih.gov/clinvar/) downloaded in November 2020 and the dbNSFP database v4.2a (https://sites.google.com/site/jpopgen/dbNSFP).

Field-specific reporting

Please select the one below that is the best fit for your research. If you are not sure, read the appropriate sections before making your selection.

☒ Life sciences ☐ Behavioural & social sciences ☐ Ecological, evolutionary & environmental sciences

For a reference copy of the document with all sections, see nature.com/documents/nr-reporting-summary-flat.pdf

Life sciences study design

All studies must disclose on these points even when the disclosure is negative.

Sample size	Sample size was determined based on number of individuals with exome data who met previously defined exclusion criteria as reported in CONSORT diagrams in the Supplementary materials
Data exclusions	Genotype, variant, and sample QC was conducted to remove low quality variants and duplicate individuals; clinical exclusions were previously defined and trait specific (see more details in Methods)
Replication	Replication of the results from the primary cohort (UK Biobank) was successfully conducted in two cohorts (TOPMed and FOURIER)
Randomization	Not relevant for analyses
Blinding	We are not aware of specific blinding taking place during central data aquisition for the UK Biobank and TOPMed; the FOURIER trial was a double-blind trial though this is not relevant to our current analysis given we performed agnostic exome-wide analyses for our included endophenotypes without utilization of prior knowledge of case status within the datasets.

Reporting for specific materials, systems and methods

We require information from authors about some types of materials, experimental systems and methods used in many studies. Here, indicate whether each material, system or method listed is relevant to your study. If you are not sure if a list item applies to your research, read the appropriate section before selecting a response.

Materials & experimental systems

n/a	Involved in the study	
☒	☐	Antibodies
☒	☐	Eukaryotic cell lines
☒	☐	Palaeontology and archaeology
☒	☐	Animals and other organisms
☐	☒	Human research participants
☒	☐	Clinical data
☒	☐	Dual use research of concern

Methods

n/a	Involved in the study	
☒	☐	ChIP-seq
☒	☐	Flow cytometry
☒	☐	MRI-based neuroimaging

Human research participants

Policy information about studies involving human research participants

Population characteristics	The UK Biobank (UKBB) is a large, national, prospective cohort of ~500,000 individuals with detailed medical history, electronic health record, and genetic data. Participants were recruited from 22 centers across the UK between 2006-2010 and aged 40-69 years at recruitment. Our analysis focused on participants with whole-exome sequencing (WES) and QT intervals extracted from resting 3-lead ECGs prior to a bicycle exercise protocol, participants with WES and LDL-C measured, and participants with WES and HbA1c measured. Please see Supplementary Tables for additional characteristics of the participant subsets.<br><br>Our LDL-C and HbA1c analyses were replicated in the Further Cardiovascular Outcomes Research With PCSK9 Inhibition in Subjects With Elevated Risk (FOURIER) trial cohort. Our QTc replication cohort included subjects from the National Heart Lung and Blood Institute's (NHLBI) Trans-Omics for Precision Medicine (TOPMed) program with whole-genome sequencing (WGS) and ECG data. The present analysis includes nine studies including the Atherosclerosis Risk in Communities (ARIC) study, Genetics of Cardiometabolic Health in the Amish (Amish), Mount Sinai BioMe Biobank (BioMe), Cleveland Family Study (CFS), Cardiovascular Health Study (CHS), Framingham Heart Study (FHS), Jackson Heart Study (JHS), Multi-Ethnic Study of Atherosclerosis (MESA), and Women's Health Initiative (WHI).
Recruitment	UK Biobank: prospective participants were invited to assessment centres in which they completed a questionnaire regarding lifestyle and medical history, underwent measurement of basic variables (blood pressure, height), and provided blood and urine samples (for biochemical measurements such as lipid levels and for preservation in order to extract DNA). A database records all disease events, drug prescriptions, and deaths for the duration of the study.

Trans-Omics in Precision Medicine (TOPMed) program: The TOPMed program is sponsored by the NHLBI with the goal of improving scientific understanding of biology underlying common diseases using integration of whole genome sequencing with molecular, imaging, environmental, and clinical data. Studies within TOPMed (community and hospital-system based) recruited a diverse pool of participants who provided biologic samples (for biochemical measurements and DNA extraction) and clinical data (including electrocardiograms).

Further Cardiovascular Outcomes Research With PCSK9 Inhibition in Subjects With Elevated Risk (FOURIER) clinical trial: this was a double-blind, randomized, placebo-controlled trial which studied the impact of adding a PCSK9-inhibitor to statin therapy on LDL-C reduction and major cardiovascular events. Participants meeting eligibility criteria were recruited from health centers across the world (https://clinicaltrials.gov/ct2/show/results/NCT01764633).

Ethics oversight

UK Biobank: Informed consent was obtained from all participants, and the UKBB received approval from the Research Ethics Committee (11/NW/0382). Our study was approved by the Mass General Brigham Human Research Committee and conducted using the UKBB Resource (Application 17488).

Trans-Omics in Precision Medicine (TOPMed) program: Molecular data for the Trans-Omics in Precision Medicine (TOPMed) program was supported by the National Heart, Lung and Blood Institute (NHLBI). Core support including read mapping and genotype calling, along with variant quality metrics and filtering were provided by the TOPMed Informatics Research Center (3R01HL-117626-02S1; contract HHSN268201800002I). Phenotype harmonization, data management, sample-identity QC, and general study coordination were provided by the TOPMed Data Coordinating Center (3R01HL-120393-02S1; contract HHSN268201800001I). Please see study specific acknowledgments in the Supplementary Materials for additional information. Use of the TOPMed cohort for analysis was approved under paper proposal ID 8472.

Further Cardiovascular Outcomes Research With PCSK9 Inhibition in Subjects With Elevated Risk (FOURIER) clinical trial: Informed consent was obtained from all participants.

Note that full information on the approval of the study protocol must also be provided in the manuscript.
